# Impact of Telemedicine-Enhanced Integrated Management of Gestational Diabetes on Pregnancy Outcomes and Glycemic Control: Real-World Study Using TangMama App

**DOI:** 10.2196/90487

**Published:** 2026-07-03

**Authors:** Jing Wang, Qunhua Wang, Yujie Liu, Rong Kang, Chenghua Li, Yixin Gong, Tian Wei, Qin Wang, Xianming Li, Xueying Zheng, Hongbo Chen, Sihui Luo, Jianping Weng

**Affiliations:** 1 Department of Endocrinology and Metabolism, The First Affiliated Hospital of USTC, University of Science and Technology of China, No. 17 Lujiang Road, Hefei, Anhui, 230001, China, 86 0551 62283691; 2 Anhui Provincial Key Laboratory of Metabolic Health and Panvascular Diseases, The First Affiliated Hospital of USTC, University of Science and Technology of China, Hefei, Anhui, China; 3 Department of Obstetrics and Gynecology, The First Affiliated Hospital of USTC, University of Science and Technology of China, Hefei, Anhui, China; 4 Department of Cardiology, The First Affiliated Hospital of USTC, University of Science and Technology of China, Hefei, Anhui, China; 5 Department of Endocrinology, Zhongda Hospital, Institute of Diabetes, School of Medicine, Southeast University, Nanjing, Jiangsu, China; 6 Department of Obstetrics and Gynecology, First Affiliated Hospital of Anhui Medical University, Hefei, Anhui, China; 7 USTC- Xunfei Healthcare Digital and Health Joint Laboratory, University of Science and Technology of China, Hefei, Anhui, China

**Keywords:** gestational diabetes, gestational diabetes mellitus, pregnancy outcomes, telemedicine, digital health, mobile phone

## Abstract

**Background:**

Gestational diabetes mellitus (GDM) is associated with substantial risks of adverse maternal and neonatal outcomes. Contemporary management approaches for GDM exhibit insufficient implementation, resulting in suboptimal glycemic control and preventable perinatal complications. The rapid evolution of mobile health technologies offers potential to enhance GDM care, yet evidence from large real-world studies remains limited.

**Objective:**

This study aimed to evaluate the impact of a telemedicine-enhanced integrated management system on pregnancy outcomes and glycemic control in women with GDM and to explore the dose-response relationship between telemedicine engagement intensity and clinical outcomes.

**Methods:**

In this real-world, prospective cohort study conducted at a provincial-level medical center in China, women with GDM were categorized into a standard care group and a telemedicine-enhanced group receiving the TangMama smartphone app in addition to standard care. We compared pregnancy outcomes and glycemic parameters between the 2 groups in an inverse probability of treatment weighting population based on propensity scores. Mediation analyses and dose-response analyses were additionally conducted to explore potential mechanisms and engagement effects.

**Results:**

A total of 4621 women with GDM were included, with 1711 in the telemedicine-enhanced group and 2910 in the standard care group. Upon inverse probability of treatment weighting analysis, the telemedicine-enhanced group demonstrated significantly lower gestational weight gain (adjusted mean difference −1.49 kg, 95% CI −1.81 to −1.17), reduced rates of excessive gestational weight gain (adjusted odds ratio [aOR] 0.61, 95% CI 0.54-0.69), cesarean section (aOR 0.80, 95% CI 0.71-0.91), hypertensive disorders in pregnancy (aOR 0.76, 95% CI 0.64-0.90), and pre-eclampsia (aOR 0.64, 95% CI 0.49-0.83). Glycemic control in the third trimester was significantly improved, with lower glycated hemoglobin A_1c_ (HbA_1c_) levels (adjusted mean difference −0.05%, 95% CI −0.08 to −0.03) and higher HbA_1c_ on-target rates. For neonatal outcomes, telemedicine-enhanced management was associated with lower rates of preterm birth (aOR 0.47, 95% CI 0.38-0.59), large-for-gestational age (aOR 0.81, 95% CI 0.69-0.96), neonatal unit admission (aOR 0.80, 95% CI 0.71-0.91), neonatal hypoglycemia (aOR 0.64, 95% CI 0.45-0.93), and neonatal hyperbilirubinemia (aOR 0.69, 95% CI 0.58-0.82). Mediation analyses identified gestational weight gain and third-trimester fasting plasma glucose as significant mediators. Higher telemedicine engagement was associated with improved glycemic control and reduced adverse outcomes in a dose-response manner.

**Conclusions:**

Telemedicine-enhanced integrated management is associated with improved maternal glycemic control and substantial reductions of adverse pregnancy outcomes among women with GDM. The observed dose-response relationship between engagement intensity and outcomes underscores the importance of promoting active patient participation. These findings support the broader integration of telemedicine into routine GDM care pathways to optimize maternal and neonatal health.

## Introduction

Gestational diabetes mellitus (GDM) elevates short- and long-term risks of adverse outcomes for both mothers and infants [1-3]. While improved glycemic control and multidisciplinary management during pregnancy have been shown to be effective in reducing the risk of perinatal complications, several barriers exist in conventional outpatient management, including time and location constraints and restricted doctor-patient communication [4,5].

Internet-based technologies hold transformative potential for improving health care accessibility in GDM management, with major guidelines explicitly endorsing telemedicine to optimize pregnancy outcomes [6]. Evidence has revealed that telemedicine interventions for GDM yield superior glycemic control, enhanced patient adherence, and reduced perinatal morbidity compared with conventional in-person care [7-9]. However, telemedicine adoption among pregnant women remains suboptimal, attributed to insufficient physician endorsement, limited awareness of benefits, perceived increase in patient burden, and distrust in virtual care efficacy [10,11]. Additionally, several limitations remain in the extant telemedicine: (1) inadequate clinical integration, where telemedicine tools operate as an isolated part rather than systematically synergizing with prenatal care protocols; (2) excessive dependence on patient-initiated data entry without timely provider interaction, resulting in incomplete clinical information and diminishing engagement; and (3) algorithmic obsolescence, as some early management approaches fail to adapt to advancing telemedicine capabilities.

Besides, China’s vast geography and uneven distribution of health care resources across its multitiered medical system have prevented advanced disease management protocols from fully reaching primary care facilities [12], posing significant challenges in delivering consistent GDM management. Despite these structural inequities, the country’s well-developed information technology infrastructure and widespread mobile phone adoption present an opportunity to practice telemedicine-based approaches that can optimize GDM management. In response to these challenges, our research team developed TangMama app, an internet-based multifunctional management platform designed based on existing evidence for women with hyperglycemia in pregnancy. This innovative mobile health app integrates seamlessly with our existing health care system. By leveraging high-quality resources of large regional medical centers, the telemedicine-enhanced integrated management synergizes hospital-based care with remote care, enabling timely patient-provider communication and continuous care delivery across hospital-home settings to optimize maternal-fetal outcomes. In this study, we evaluate the impact of this telemedicine-enhanced integrated care model on pregnancy outcomes and maternal glycemic control in women with GDM and further explore the relationship between different levels of patient engagement with the app and subsequent pregnancy outcomes.

## Methods

### Study Design and Participants

This study is a real-world, prospective cohort study conducted at Anhui Provincial Hospital, a provincial-level regional comprehensive medical center in East China. As the headquarters, Anhui Provincial Hospital connects more than 80 primary care facilities in an established medical consortium and has capabilities in managing pregnancies with complications. In addition, as the leading center, Anhui Provincial Hospital and the other 65 hospitals have established the Anhui Province Gestational Diabetes Specific Alliance dedicated to improving perinatal care for women with hyperglycemia [13].

Our study population was enrolled from women aged 18-55 years, with GDM, and singleton pregnancy, who attended Anhui Provincial Hospital between January 1, 2022, and August 1, 2025. GDM was diagnosed between 24 and 28 weeks of gestation using the World Health Organization 2013 criteria [14]. The exclusion criteria were pre-existing diabetes and complications involving significant organ diseases. The detailed eligibility criteria are described in “eMethods” in Multimedia Appendix 1.

### Telemedicine-Integrated GDM Management

The telemedicine-based GDM care is implemented through the TangMama app in addition to standard care, with the connection of its cloud platform. TangMama app mainly offered the following modules of function: (1) patient monitoring and data synchronization—continuous longitudinal tracking of key health metrics, with physicians accessing patients’ real-time out-of-hospital data via doctor terminal platform; (2) automated data upload—besides manual entry, monitoring data can be automatically collected and transmitted to the system back end when using 5G-enabled smart devices (including Bluetooth-connected glucometers, weight scales, and blood pressure monitors) at patients’ disposal; (3) intelligent alerts and reminders based on input or uploaded metrics—both the patient interface and the TangMama care team portal automatically generated alerts when abnormal data patterns were detected, assisting patients in promptly identifying concerning indicators that required medical attention, while also reminding them to schedule timely in-person clinical visits when necessary; (4) interactive care and personalized medicine support—one-to-one medical consultations to receive timely personalized medical advice regarding glycemic control, exercise, nutrition, and emotional support from the TangMama care team; and (5) structured education—59 educational courses inspected by endocrinologists and obstetricians tailored to the characteristics of different gestational stages. Figures S1 and S2 in Multimedia Appendix 1 show the screenshots of the TangMama platform.

**Figure 1.**
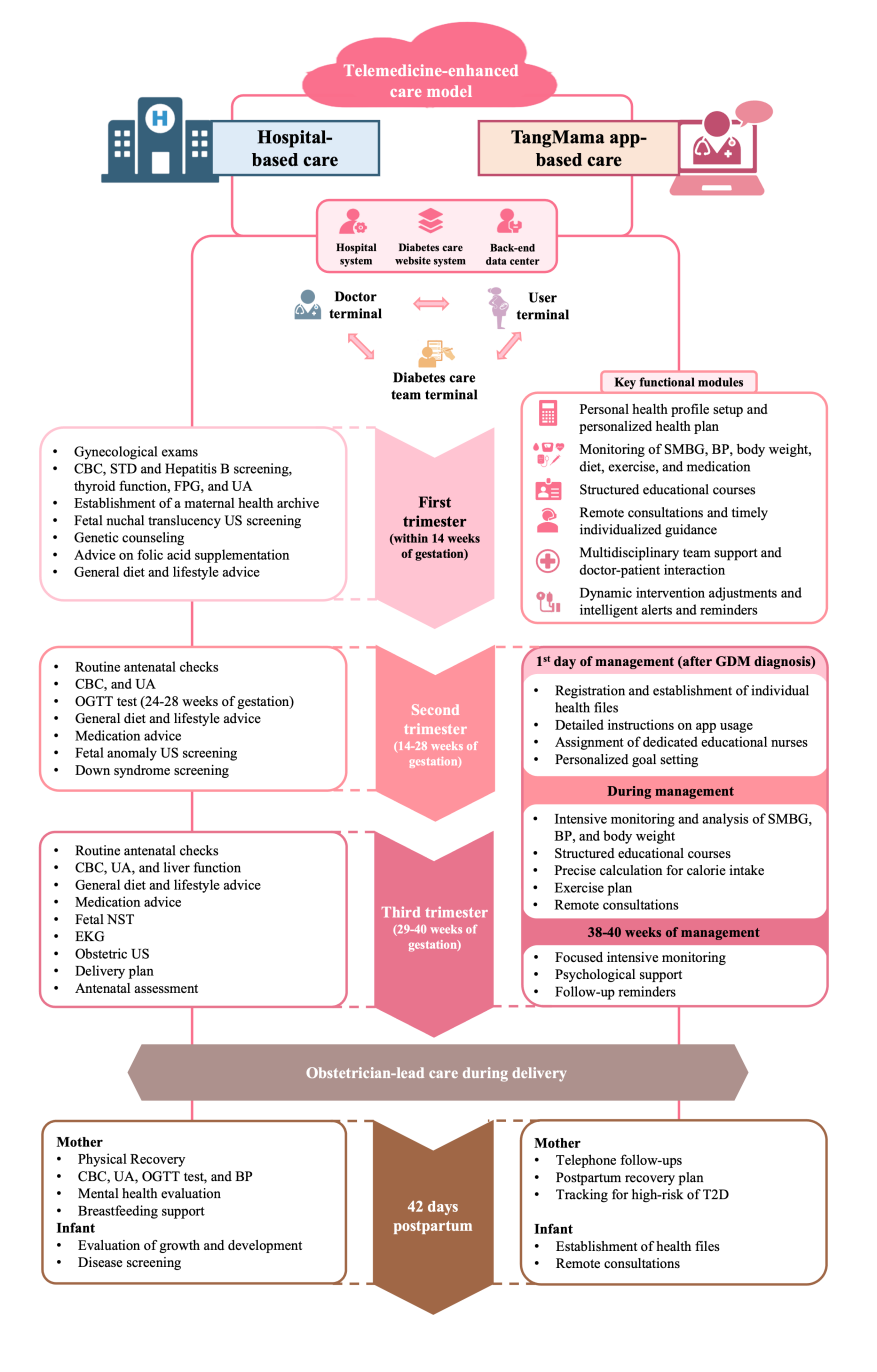
The workflow of the telemedicine-enhanced integrated care model for GDM. This figure depicts the integration of the telemedicine-enhanced care for women with GDM into the Chinese nationwide standard clinical antenatal care pathway based on the guidance of the World Health Organization. The left side shows the standard hospital-based obstetric care pathway throughout pregnancy, while the right side illustrates the additional TangMama app-based remote monitoring, education, consultations, and multidisciplinary support that begin after GDM diagnosis. A routine antenatal check includes measurement of maternal height, weight, blood pressure, uterus fundal height, and abdominal circumference; assessment of edema; and measurement of fetal heart rate. BP: blood pressure; CBC: complete blood count; EKG: electrocardiogram; FPG: fasting plasma glucose; GDM: gestational diabetes mellitus; NST: nonstress test; OGTT: oral glucose tolerance test; SMBG: self-monitoring of blood glucose; STD: sexually transmitted disease; T2D: type 2 diabetes; UA: urinalysis; US: ultrasound.

The TangMama cloud platform connects hospital information systems with an out-of-hospital diabetes care web system, creating a unified network linking 3 essential terminals: the patient interface (user terminal), the health care provider interface (doctor terminal), and the specialized education team platform (diabetes care team terminal). The TangMama care team comprises various health care professionals, including endocrinologists, obstetricians, gynecologists, diabetes education nurses, qualified dietitians, yoga instructors, and psychological counselors, all collaborating to provide multidisciplinary GDM management throughout pregnancy. Figure 1 illustrates the workflow of the telemedicine-enhanced integrated care model for GDM.

Although the TangMama platform was technically launched in 2021 and has been designated as the standardized management tool within the Anhui Province Gestational Diabetes Specific Alliance, its actual uptake in routine care remained low prior to this study. Telemedicine-enhanced management had not been systematically integrated into standard antenatal care pathways. Therefore, this study evaluates the real-world impact of actively introducing and integrating the TangMama app-based telemedicine-enhanced care into routine clinical practice, rather than assessing an already fully implemented routine intervention.

### Patient Grouping

Group assignment was not randomized; instead, it was based on patients’ voluntary self-selection to use the TangMama app. Specifically, following GDM diagnosis, all patients were promptly referred to a specialized outpatient diabetes care clinic for diabetes evaluation and education. Moreover, health care professionals would introduce and assist patients in downloading the TangMama app for telemedicine-enhanced management.

Eligible women with documented app usage data after GDM diagnosis during their current pregnancy and who were linked to a health care provider at Anhui Provincial Hospital were regarded as the telemedicine-enhanced group. Upon their use of the TangMama app, we collected their app usage data from the TangMama back-end database, which comprised 3 key behavioral indicators: the number of self-monitoring of blood glucose (SMBG) records uploaded, teleconsultation frequency, and completion rates of structured educational courses. We collected their sociodemographic information, clinical data, and pregnancy outcomes via face-to-face interviews, the electronic health record system in the centers of the Anhui Province Gestational Diabetes Specific Alliance, and the Maternal and Child Health Care Information System of China [15]. No participant in this study had access to TangMama-based telemedicine care prior to GDM diagnosis, and app usage data recorded before the date of diagnosis were not included in the analysis.

Other eligible participants managed in the study center during the same time period, who did not register with or use the TangMama app for any reason, were considered as the standard care group. We followed them in the outpatient clinic and collected their information through face-to-face interviews, as well as the electronic health record system and the Maternal and Child Health Care Information System.

All the women studied received standard clinical prenatal care, which was implemented according to the Chinese maternal health care infrastructure guidelines and developed based on the World Health Organization recommendations for routine maternity care [16]. During regularly scheduled prenatal visits, health care providers conducted comprehensive evaluations of these patients’ clinical status and disease management while making necessary adjustments to their management plans based on individual therapeutic responses and pregnancy progression. And the women in the telemedicine-enhanced group were provided with telemedicine-based care implemented through the TangMama app in addition to standard care.

### Outcomes and Definitions

Outcome measures were categorized into maternal outcomes and neonatal outcomes. Maternal outcomes encompassed gestational weight gain (GWG), excessive gestational weight gain (EGWG) [17], gestational age at delivery, mode of delivery (cesarean section, normal vaginal, or forceps), episiotomy, hypertensive disorders in pregnancy, pre-eclampsia, shoulder dystocia, and pregnancy loss (including miscarriage and stillbirth). Maternal glycemic control was evaluated using mean fasting plasma glucose (FPG) in the third trimester, glycated hemoglobin A_1c_ (HbA_1c_) level in the third trimester, and HbA_1c_ on-target rate. Mean FPG in the third trimester was defined as the mean value of at least 3 FPG measurements taken within 1 week prior to delivery. HbA_1c_ in the third trimester was measured within 1 month prior to delivery. Neonatal outcomes were evaluated through preterm birth, large-for-gestational age (LGA), macrosomia, small-for-gestational age (SGA), low birth weight (LBW), Apgar scores at 1 and 5 minutes, and neonatal unit admission. Complications monitored included neonatal hypoglycemia, hyperbilirubinemia, respiratory distress, congenital heart defects, and neonatal death. Outcomes were defined according to previous reports (Table S1 in Multimedia Appendix 1) [18,19].

### Ethical Considerations

This study was approved by the Ethics Committee of the Anhui Provincial Hospital (number 2021-KY-LS [241]) and registered on the Chinese Clinical Trial Registry (registration number ChiCTR2000030972). The study has been performed under the guidelines of the Declaration of Helsinki. Written informed consent was obtained from all participants after GDM diagnosis and prior to app introduction. No compensation was provided to participants for their participation in this study.

### Statistical Analysis

To address potential selection bias, inverse probability of treatment weighting (IPTW) was implemented to balance baseline characteristics between groups [20]. Maternal age, education level, parity, prepregnancy BMI, 1-hour postload glucose on the oral glucose tolerance test (OGTT), and insulin treatment were included in the propensity score (PS) calculation. Although insulin treatment is typically initiated following GDM diagnosis, it was included as a proxy for underlying GDM severity and clinical disease burden to ensure adequate adjustment for baseline differences in metabolic status between groups. Standardized mean differences were calculated for each covariate, with standardized mean difference ≤0.1 after IPTW indicating adequate balance between groups. Binary outcomes were analyzed using IPTW-adjusted logistic regression models, yielding odds ratios and 95% CIs, while continuous outcomes were evaluated using IPTW-adjusted linear regression models to obtain mean differences with 95% CIs.

We conducted several sensitivity analyses to validate the robustness of our findings. To minimize potential bias arising from including insulin treatment as a postbaseline variable, we conducted a sensitivity analysis using IPTW based on a PS model excluding insulin treatment. We then implemented propensity score matching to create balanced comparison groups, with the standard care group matched 1:1 to the telemedicine-enhanced group using a nearest neighbor approach with a caliper width of 0.02. We also conducted analyses restricted to nulliparous participants to address potential confounding effects of parity. To address potential timing bias, we performed an additional analysis that excluded participants who registered with the TangMama app after 28 weeks of gestation.

In exploratory analyses, we examined potential mechanistic pathways through which telemedicine influences pregnancy outcomes. Using the bootstrap method, we investigated whether GWG and mean FPG in the third trimester mediated the relationship between telemedicine-enhanced care and various pregnancy outcomes [21,22]. Moreover, we evaluated the dose-response relationship between telemedicine engagement intensity and pregnancy outcomes. Engagement intensity was quantified using a composite score derived from the 3 behavioral indicators of app engagement: the number of SMBG records uploaded, teleconsultation frequency, and the completion rates of structured educational courses. These indicators were combined into a composite score using the Criteria Importance Through Intercriteria Correlation method [23], which determines objective weights based on the contrast intensity of each indicator (measured by standard deviation) and the degree of conflict among indicators (measured by their intercorrelations). SMBG records accounted for 32.86% of the total weight, teleconsultation frequency for 37.24%, and completion rates of structured educational courses for 29.90%. The composite engagement score for each participant was subsequently computed as the weighted sum of the 3 standardized indicator values, with higher scores indicating greater engagement intensity. Within the telemedicine-enhanced group, patients were stratified into tertiles based on the generated composite engagement scores: low (0‐7.819), moderate (7.820‐45.996), and high (46.050‐723.297) engagement. Multivariate logistic regression was used to examine gradient associations across engagement groups. Multiple imputations were used to handle missing values in all analyses. Statistical analyses were performed using R software (version 4.4.2; R Foundation for Statistical Computing). Statistical significance was taken at *P*<.05.

## Results

### Baseline Characteristics

The final analysis included 4621 pregnant women with GDM, comprising 1711 (37.0%) patients in the telemedicine-enhanced group and 2910 (63%) patients in the standard care group (Figure S3 in Multimedia Appendix 1). Baseline maternal characteristics before and after IPTW adjustment are presented in Table 1. The IPTW population had a mean age of 31.8 (SD 4.1) years and a mean prepregnancy BMI of 23.1 (SD 3.4) kg/m^2^. Prior to propensity adjustment, significant differences between the telemedicine-enhanced and standard care groups were observed in 3 characteristics: the prevalence of nulliparous women (63.1% vs 56.4%; *P*<.001), metformin treatment (0.7% vs 0.2%; *P*=.02), and 1-hour postload glucose on OGTT (10.05 mmol/L vs 9.92 mmol/L; *P*=.02). All other baseline covariates showed no statistically significant differences between groups (*P*>.05). Following IPTW adjustment, an excellent balance of baseline characteristics was achieved between the study groups.

**Table 1.** Baseline characteristics of study participants^a^.

Characteristics	Before IPTW^b^					After IPTW			
	Overall (N=4621)	Telemedicine-enhanced group (n=1711)	Standard care group (n=2910)	*P* value	SMD^c^	Telemedicine-enhanced group (n=1709.7)	Standard care group (n=2910.6)	*P* value	SMD
Age (years), mean (SD)	31.8 (4.1)	32.0 (4.1)	31.8 (4.2)	.17	0.042	31.9 (4.0)	31.9 (4.2)	.83	0.006
Education level, n (%)									
High school or lower	904 (19.6)	310 (18.1)	594 (20.4)	.06	0.058	334.3 (19.6)	568.7 (19.5)	.99	<0.001
College or higher	3717 (80.4)	1401 (81.9)	2316 (79.6)			1375.5 (80.4)	2341.9 (80.5)		
Nulliparous, n (%)	2720 (58.9)	1080 (63.1)	1640 (56.4)	<.001	0.138	1010.4 (59.1)	1715.2 (58.9)	.91	0.003
Anthropometric indices, mean (SD)									
Height, m	1.61 (0.05)	1.62 (0.05)	1.61 (0.05)	.53	0.019	1.62 (0.05)	1.61 (0.05)	.56	0.018
Prepregnancy weight, kg	60.3 (9.7)	60.6 (9.4)	60.1 (10.0)	.10	0.051	60.3 (9.3)	60.3 (10.1)	.81	0.007
Prepregnancy BMI, kg/m^2^	23.1 (3.4)	23.2 (3.3)	23.0 (3.5)	.06	0.059	23.1 (3.3)	23.1 (3.5)	.94	0.002
Systolic BP^d^, mm Hg	123.6 (13.3)	123.2 (12.4)	123.8 (13.8)	.11	0.050	123.5 (12.4)	123.6 (13.7)	.89	0.004
Diastolic BP, mm Hg	79.2 (10.4)	78.8 (9.7)	79.4 (10.7)	.07	0.055	78.9 (9.8)	79.2 (10.6)	.31	0.031
FPG^e^ in the first trimester, mmol/L	4.85 (0.61)	4.85 (0.61)	4.85 (0.60)	.72	0.011	4.85 (0.61)	4.85 (0.60)	.83	0.007
Values on 75-g OGTT^f^, mmol/L, mean (SD)									
0 minutes	5.07 (0.66)	5.06 (0.63)	5.08 (0.68)	.27	0.034	5.06 (0.63)	5.08 (0.68)	.38	0.027
60 minutes	9.97 (1.66)	10.05 (1.59)	9.92 (1.69)	.02	0.075	9.97 (1.61)	9.97 (1.68)	.99	<0.001
120 minutes	8.41 (1.64)	8.45 (1.62)	8.38 (1.65)	.19	0.040	8.41 (1.61)	8.39 (1.65)	.75	0.010
Pre-existing hypertension	42 (0.9)	14 (0.8)	28 (1.0)	.74	0.015	13.7 (0.8)	28.2 (1.0)	.56	0.018
Management of diagnosis, mean (SD)									
Lifestyle intervention	4371 (94.6)	1622 (94.8)	2749 (94.5)	.68	0.015	1614.2 (94.4)	2753.1 (94.6)	.81	0.008
Insulin injection	232 (5.0)	77 (4.5)	155 (5.3)	.24	0.038	88.9 (5.2)	148.1 (5.1)	.88	0.005
Metformin	18 (0.4)	12 (0.7)	6 (0.2)	.02	0.074	6.7 (0.4)	11.1 (0.4)	.97	0.001

a Continuous variables are presented as the mean (SD), and categorical variables are expressed as n (%). Maternal age, education level, parity, prepregnancy BMI, 1-hour postload glucose on the OGTT, and insulin treatment were included in the propensity score model for IPTW.

b IPTW: inverse probability of treatment weighting.

c SMD: standardized mean difference.

d BP: blood pressure.

e FPG: fasting plasma glucose.

f OGTT: oral glucose tolerance test.

### Maternal and Neonatal Outcomes

Multivariable analyses indicated that significant differences in maternal outcomes were observed between the telemedicine-enhanced and standard care groups after IPTW adjustment (Table 2). The telemedicine-enhanced group demonstrated lower GWG (adjusted mean difference [aMD] −1.49 kg, 95% CI −1.81 to −1.17; *P*<.001) and reduced rates of several complications: EGWG (adjusted odds ratio [aOR] 0.61, 95% CI 0.54-0.69; *P*<.001), cesarean section (aOR 0.80, 95% CI 0.71-0.91; *P*<.001), hypertensive disorders in pregnancy (aOR 0.76, 95% CI 0.64-0.90; *P*=.002), pre-eclampsia (aOR 0.64, 95% CI 0.49-0.83; *P*=.001), and pregnancy loss (aOR 0.22, 95% CI 0.08-0.63; *P*=.005). No significant differences were observed between groups in the rates of forceps delivery, episiotomy, or shoulder dystocia. While modest in absolute terms, the telemedicine-enhanced group showed statistically significant improvements in glycemic control parameters. After adjusting for maternal age, education level, parity, prepregnancy BMI, 1-hour postload glucose on OGTT, insulin treatment, and gestational age, the telemedicine-enhanced group showed improved glycemic control in the third trimester. Specifically, HbA_1c_ levels were lower (aMD −0.05%, 95% CI −0.08 to −0.03; *P*<.001), along with reduced mean FPG values (aMD −0.07 mmol/L, 95% CI −0.10 to −0.03; *P*<.001). The telemedicine-enhanced group also achieved higher HbA_1c_ on-target rates for both thresholds (<6%, aOR 1.23, 95% CI 1.04-1.46; *P*=.02; <5.6%, aOR 1.27, 95% CI 1.12-1.44; *P*<.001).

Neonatal outcomes also showed substantial improvements in the telemedicine-enhanced group compared with the standard care group (Table 3). Significant reductions were observed in the rates of multiple adverse outcomes: preterm birth (aOR 0.47, 95% CI 0.38-0.59; *P*<.001), LGA (aOR 0.81, 95% CI 0.69-0.96; *P*=.02), LBW (aOR 0.39, 95% CI 0.29-0.52; *P*<.001), and compromised Apgar scores at both 1 minute (aOR 0.41, 95% CI 0.22-0.77; *P*=.006) and 5 minutes (aOR 0.17, 95% CI 0.04-0.71; *P*=.02). Lower risks for neonatal unit admission (aOR 0.80, 95% CI 0.71-0.91; *P*<.001) and neonatal complications including hypoglycemia (aOR 0.64, 95% CI 0.45-0.93; *P*=.02), hyperbilirubinemia (aOR 0.69, 95% CI 0.58-0.82; *P*<.001), respiratory distress (aOR 0.34, 95% CI 0.20-0.56; *P*<.001), and congenital heart defects (aOR 0.76, 95% CI 0.66-0.87; *P*<.001) were observed in the telemedicine-enhanced group. No significant differences were found in the rates of macrosomia, SGA, or neonatal death.

**Table 2.** The association between telemedicine-enhanced group and maternal outcomes in comparison with standard care group^a^.

Maternal outcomes	Before IPTW^b^						After IPTW			
	Telemedicine-enhanced group (n=1711)	Standard care group (n=2910)	Crude OR^c^ or mean difference (95% CI)	*P* value	Adjusted OR or mean difference (95% CI)	*P* value	Crude OR or mean difference (95% CI)	*P* value	Adjusted OR or mean difference (95% CI)	*P* value
GWG^d^, kg	11.6 (5.3)	13.2 (5.7)	−1.59 (−1.93 to −1.26)^e^	<.001	−1.49 (−1.82 to −1.16)^e^	<.001	−1.50 (−1.83 to −1.17)^e^	<.001	−1.49 (−1.81 to −1.17)^e^	<.001
EGWG^f^	589 (34.4)	1572 (54.0)	0.62 (0.54 to 0.70)^g^	<.001	0.61 (0.54 to 0.69)^g^	<.001	0.62 (0.55 to 0.70)^g^	<.001	0.61 (0.54 to 0.69)^g^	<.001
Gestational age at delivery (weeks)	39.0 (1.5)	38.5 (2.1)	0.45 (0.34 to 0.57)^g^	<.001	0.42 (0.31 to 0.53)^g^	<.001	0.43 (0.32 to 0.53)^g^	<.001	0.43 (0.32 to 0.53)^g^	<.001
Mode of delivery										
Cesarean section	921 (53.8)	1710 (58.8)	0.82 (0.73 to 0.92)^g^	.001	0.81 (0.71 to 0.92)^g^	.001	0.82 (0.72 to 0.92)^g^	.001	0.80 (0.71 to 0.91)^g^	<.001
Normal vaginal delivery	775 (45.3)	1177 (40.4)	1.22 (1.08 to 1.38)^g^	.001	1.23 (1.09 to 1.40)^g^	.001	1.22 (1.08 to 1.38)^g^	.001	1.24 (1.10 to 1.40)^g^	<.001
Forceps delivery	15 (0.9)	23 (0.8)	1.11 (0.57 to 2.11)^g^	.75	1.09 (0.55 to 2.09)^g^	.79	1.03 (0.53 to 1.99)^g^	.93	1.07 (0.55 to 2.08)^g^	.84
Episiotomy	107 (6.3)	178 (6.1)	1.02 (0.80 to 1.31)^g^	.85	0.93 (0.72 to 1.19)^g^	.56	0.93 (0.72 to 1.20)^g^	.58	0.93 (0.72 to 1.20)^g^	.57
Hypertensive disorders in pregnancy	263 (15.4)	528 (18.1)	0.82 (0.70 to 0.96)^g^	.02	0.77 (0.65 to 0.91)^g^	.002	0.77 (0.65 to 0.90)^g^	.001	0.76 (0.64 to 0.90)^g^	.002
Pre-eclampsia	84 (4.9)	208 (7.1)	0.67 (0.51 to 0.87)^g^	.003	0.64 (0.49 to 0.83)^g^	.001	0.63 (0.49 to 0.83)^g^	<.001	0.64 (0.49 to 0.83)^g^	.001
Shoulder dystocia	2 (0.1)	7 (0.2)	0.49 (0.07 to 2.01)^g^	.37	0.49 (0.07 to 1.87)^g^	.32	0.43 (0.09 to 2.08)^g^	.29	0.43 (0.09 to 2.07)^g^	.29
Pregnancy loss	4 (0.2)	30 (1.0)	0.22 (0.07 to 0.57)^g^	.005	0.24 (0.07 to 0.61)^g^	.008	0.22 (0.08 to 0.62)^g^	.004	0.22 (0.08 to 0.63)^g^	.005
Parameters of glycemic control										
HbA_1c_^h^ in the third trimester, %	5.57 (0.41)	5.62 (0.43)	−0.05 (−0.08 to −0.02)^i^	<.001	−0.05 (−0.08 to −0.03)^i^	<.001	−0.05 (−0.08 to −0.03)^i^	<.001	−0.05 (−0.08 to −0.03)^i^	<.001
HbA_1c_ in the third trimester <6%	1446 (84.5)	2378 (81.7)	1.22 (1.04 to 1.44)^g^	.02	1.23 (1.04 to 1.46)^g^	.01	1.23 (1.05 to 1.45)^g^	.01	1.23 (1.04 to 1.46)^g^	.02
HbA_1c_ in the third trimester <5.6%	938 (54.8)	1453 (49.9)	1.22 (1.08 to 1.37)^g^	.001	1.27 (1.12 to 1.44)^g^	<.001	1.25 (1.10 to 1.40)^g^	<.001	1.27 (1.12 to 1.44)^g^	<.001
Mean FPG^j^ in the third trimester, mmol/L	4.76 (0.54)	4.82 (0.69)	−0.06 (−0.10 to −0.03)^k^	.001	−0.06 (−0.10 to −0.03)^k^	<.001	−0.07 (−0.10 to −0.03)^k^	<.001	−0.07 (−0.10 to −0.03)^k^	<.001

a Continuous variables are presented as the mean (SD), and categorical variables are expressed as n (%).

b IPTW: inverse probability of treatment weighting.

c OR: odds ratio.

d GWG: gestational weight gain.

e The mean difference is estimated from a linear regression model; in the adjusted analysis, the adjusted mean difference was adjusted for maternal age, education level, parity, prepregnancy BMI, 1-hour postload glucose on oral glucose tolerance test, and insulin treatment.

f EGWG: excessive gestational weight gain.

g The OR is estimated from a logistic regression model; in the adjusted analysis, the adjusted OR was adjusted for maternal age, education level, parity, prepregnancy BMI, 1-hour postload glucose on oral glucose tolerance test, and insulin treatment.

h HbA_1c_ : glycated hemoglobin A_1c_.

i The OR is estimated from a logistic regression model; in the adjusted analysis, the adjusted OR was adjusted for maternal age, education level, parity, prepregnancy BMI, 1-hour postload glucose on oral glucose tolerance test, insulin treatment, and gestational age.

j FPG: fasting plasma glucose.

k The mean difference is estimated from a linear regression model; in the adjusted analysis, the adjusted mean difference was adjusted for maternal age, education level, parity, prepregnancy BMI, 1-hour postload glucose on oral glucose tolerance test, insulin treatment, and gestational age.

**Table 3.** The association between telemedicine-enhanced group and neonatal outcomes in comparison with standard care group^a^.

Neonatal outcomes	Before IPTW^b^						After IPTW			
	Telemedicine-enhanced group (n=1711)	Standard care group (n=2910)	Crude OR^c^ or mean difference (95% CI)	*P* value	Adjusted OR or mean difference (95% CI)	*P* value	Crude OR or mean difference (95% CI)	*P* value	Adjusted OR or mean difference (95% CI)	*P* value
Preterm birth	128 (7.5)	424 (14.6)	0.47 (0.38-0.58)^d^	<.001	0.48 (0.39-0.59)^d^	<.001	0.48 (0.39-0.60)^d^	<.001	0.47 (0.38-0.59)^d^	<.001
LGA^e^_,_^f^	260 (15.2)	519 (18.0)	0.82 (0.69-0.96)^d^	.02	0.83 (0.70-0.98) ^d^	.03	0.82 (0.69-0.96)^d^	.02	0.81 (0.69-0.96)^d^	.02
Macrosomia^f^	139 (8.1)	259 (9.0)	0.90 (0.72-1.11)^d^	.32	0.89 (0.72-1.11)^d^	.31	0.88 (0.71-1.09)^d^	.25	0.89 (0.71-1.11)^d^	.30
SGA^g^_,_^f^	89 (5.2)	176 (6.1)	0.85 (0.65-1.10)^d^	.21	0.83 (0.63-1.07)^d^	.16	0.83 (0.64-1.08)^d^	.16	0.83 (0.64-1.08)^d^	.16
LBW^h^_,_^f^	62 (3.6)	252 (8.8)	0.39 (0.29-0.52)^d^	<.001	0.39 (0.29-0.52)^d^	<.001	0.39 (0.30-0.53)^d^	<.001	0.39 (0.29-0.52)^d^	<.001
Birth weight, kg^f^	3.31 (0.49)	3.24 (0.59)	0.07 (0.04-0.10)^i^	<.001	0.07 (0.04-0.10)^i^	<.001	0.07 (0.04-0.10)^i^	<.001	0.07 (0.04-0.10)^i^	<.001
Birth length, cm^f^	50.0 (1.9)	49.6 (2.6)	0.47 (0.33-0.62)^i^	<.001	0.47 (0.33-0.62)^i^	<.001	0.47 (0.34-0.60)^i^	<.001	0.47 (0.34-0.60)^i^	<.001
Apgar score <7 at 1 minute^f^	12 (0.7)	48 (1.7)	0.42 (0.21-0.76)^d^	.007	0.41 (0.21-0.75)^d^	.006	0.41 (0.22-0.77)^d^	.006	0.41 (0.22-0.77)^d^	.006
Apgar score <7 at 5 minutes^f^	2 (0.1)	19 (0.7)	0.18 (0.03-0.61)^d^	.02	0.18 (0.03-0.62)^d^	.02	0.17 (0.04-0.71)^d^	.02	0.17 (0.04-0.71)^d^	.02
Neonatal unit admission^f^	698 (40.8)	1315 (45.2)	0.84 (0.74-0.94)^d^	<.001	0.80 (0.71-0.91)^d^	<.001	0.81 (0.71-0.91)^d^	<.001	0.80 (0.71-0.91)^d^	<.001
Neonatal hypoglycemia^f^	43 (2.5)	113 (3.9)	0.64 (0.44-0.90)^d^	.01	0.64 (0.45-0.92)^d^	.02	0.64 (0.45-0.92)^d^	.02	0.64 (0.45-0.93)^d^	.02
Neonatal hyperbilirubinemia^f^	205 (12.0)	473 (16.3)	0.70 (0.59-0.84)^d^	<.001	0.70 (0.59-0.82)^d^	<.001	0.69 (0.58-0.82)^d^	<.001	0.69 (0.58-0.82)^d^	<.001
Neonatal respiratory distress^f^	18 (1.1)	94 (3.2)	0.32 (0.19-0.52)^d^	<.001	0.33 (0.19-0.53)^d^	<.001	0.34 (0.20-0.56)^d^	<.001	0.34 (0.20-0.56)^d^	<.001
Congenital heart defects^f^	408 (23.8)	830 (28.5)	0.78 (0.68-0.90)^d^	<.001	0.76 (0.66-0.88)^d^	<.001	0.76 (0.66-0.88)^d^	<.001	0.76 (0.66-0.87)^d^	<.001
Neonatal death^f^	1 (0.1)	4 (0.1)	0.42 (0.02-2.87)^d^	.44	0.46 (0.02-3.22)^d^	.49	0.40 (0.04-3.59)^d^	.41	0.43 (0.05-3.86)^d^	.45

a Continuous variables are presented as the mean (SD), and categorical variables are expressed as n (%).

b IPTW: inverse probability of treatment weighting;

c OR, odds ratio.

d The OR is estimated from a logistic regression model; in the adjusted analysis, the adjusted OR was adjusted for maternal age, education level, parity, prepregnancy BMI, 1-hour postload glucose on oral glucose tolerance test, and insulin treatment.

e LGA: large-for-gestational age.

f Thirty-four pregnancy losses were excluded from the analysis (4 in the telemedicine-enhanced group and 30 in the standard care group).

g SGA: small-for-gestational age,

h LBW: low birth weight.

i The mean difference is estimated from a linear regression model; in the adjusted analysis, the adjusted mean difference was adjusted for maternal age, education level, parity, prepregnancy BMI, 1-hour postload glucose on oral glucose tolerance test, and insulin treatment.

### Sensitivity Analyses

Across all supplementary analyses, including the alternative IPTW model excluding insulin treatment from the PS (Table S2 in Multimedia Appendix 1), propensity score matching population (Table S3 in Multimedia Appendix 1), nulliparous patients (Table S4 in Multimedia Appendix 1), and the population that excluded those who registered with TangMama app after 28 weeks of gestation (Table S5 in Multimedia Appendix 1), the beneficial associations between telemedicine-enhanced and maternal-neonatal outcomes remained largely consistent with our primary findings, thereby strengthening the credibility of our main analysis.

### Mediation of Telemedicine-Enhanced Care and Pregnancy Outcomes Through GWG and Blood Glucose Levels

Figure S4 in Multimedia Appendix 1 summarizes the potential mediating effects of GWG and mean FPG in the third trimester between telemedicine-enhanced care and various pregnancy outcomes. Telemedicine-enhanced care exerted substantial mediating effects through GWG on several key outcomes, with mediation proportions of 37.24% for cesarean section, 22.59% for pre-eclampsia, and a particularly strong 81.01% for LGA births. Mean FPG in the third trimester demonstrated significant but generally smaller mediating effects between telemedicine-enhanced care and multiple outcomes, including LGA (12.86%), cesarean section (3.38%), preterm birth (1.92%), neonatal hypoglycemia (5.06%), and congenital heart defects (5.12%). These findings suggest that the beneficial effects of telemedicine-enhanced care on pregnancy outcomes are partially mediated through optimized GWG and improved glycemic control.

### Dose-Response Relationships Between App Engagement Intensity and Pregnancy Outcomes

**Figure 2.**
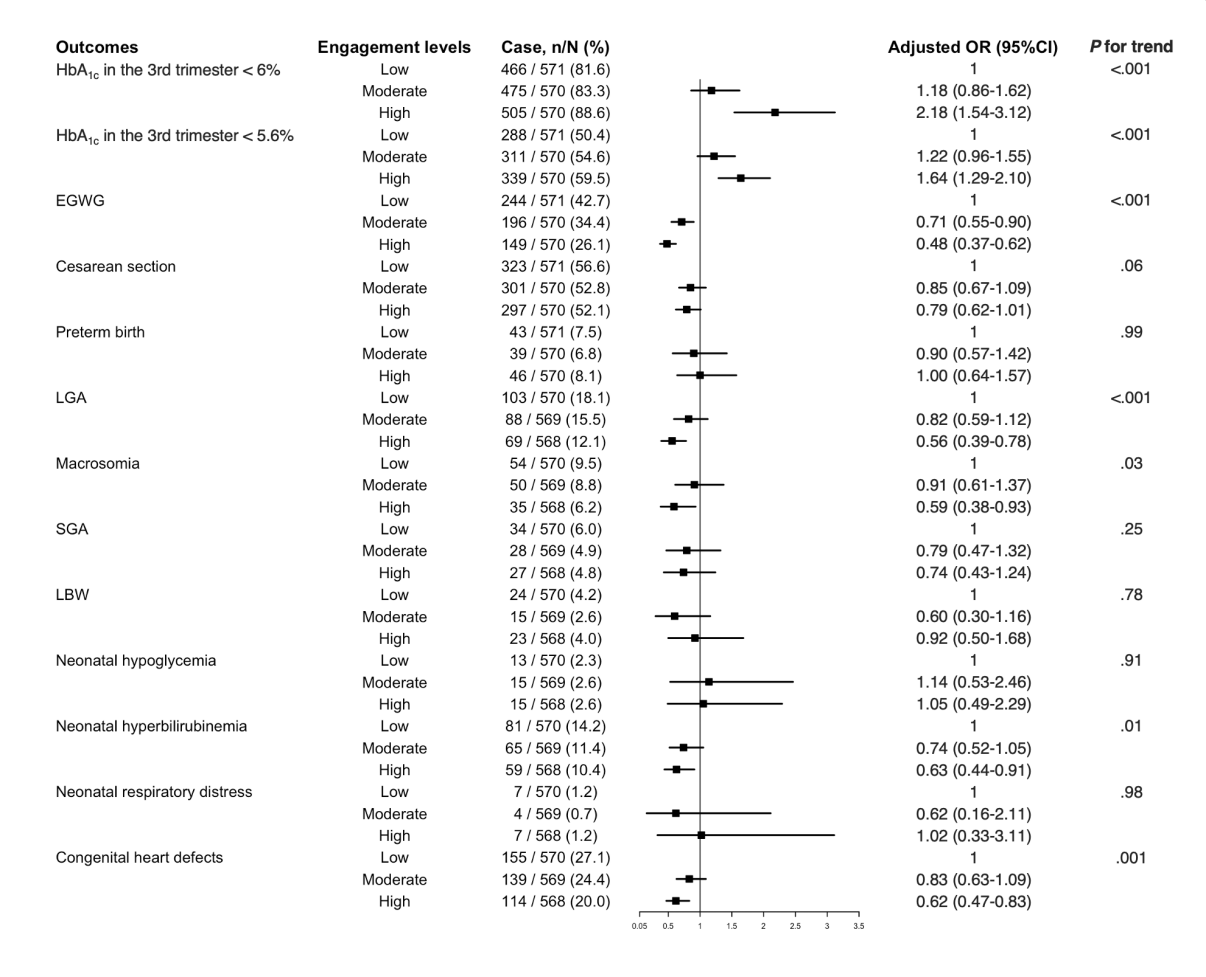
Multivariate logistic regression analysis for the association between telemedicine engagement levels and pregnancy outcomes. The OR is estimated from a logistic regression model; in the adjusted analysis, the adjusted OR was adjusted for maternal age, education level, parity, prepregnancy BMI, 1-hour postload glucose on oral glucose tolerance test, and insulin treatment. Four stillbirths were excluded from the analysis. CI: confidence interval; EGWG: excessive gestational weight gain; HbA_1c_,: glycated hemoglobin A_1c_; LBW: low birth weight; LGA: large-for-gestational age; OR: odds ratio; SGA: small-for-gestational age.

After adjusting for confounding factors, higher telemedicine engagement was significantly associated with improved glycemic control and reduced adverse outcomes (Figure 2). Enhanced telemedicine engagement correlated with increased HbA_1c_ on-target rates at both the 6% (aOR 2.18, 95% CI 1.54-3.12; *P* value for trend <.001) and the 5.6% threshold (aOR 1.64, 95% CI 1.29-2.10; *P* value for trend <.001). Moreover, higher engagement exhibited protective associations against EGWG (aOR 0.48, 95% CI 0.37-0.62; *P* value for trend <.001), LGA (aOR 0.56, 95% CI 0.39-0.78; *P* value for trend <.001), macrosomia (aOR 0.59, 95% CI 0.38-0.93; *P* value for trend=.03), and neonatal complications, specifically neonatal hyperbilirubinemia (aOR 0.63, 95% CI 0.44-0.91; *P* value for trend=.01) and congenital heart defects (aOR 0.62, 95% CI 0.47-0.83; *P* value for trend=.001).

## Discussion

### Principal Findings

In this real-world study, we evaluated the impact of a telemedicine-enhanced integrated management program that combines hospital resources with efficient remote services, offering a new workflow that strengthens remote communication and assistance between patients and health care providers while promoting personalized management. In patients with GDM, we observed that telemedicine-enhanced integrated management was significantly associated with improved various pregnancy outcomes: maternal outcomes including GWG, cesarean section, pre-eclampsia, maternal glycemic control, and neonatal outcomes including preterm birth, LGA, LBW, neonatal hypoglycemia, hyperbilirubinemia, respiratory distress, and congenital heart defects. These findings suggest that this approach can further optimize comprehensive prenatal care, offering important implications for protecting reproductive health in the childbearing population and enhancing overall birth outcomes.

Our results differ from those reported by Mackillop et al [24], who found lower cesarean section rates in their app intervention group but no significant differences in blood glucose levels or other adverse pregnancy outcomes. This discrepancy might be explained by their later intervention initiation (mean gestational age of 31 weeks), which likely provided insufficient time for the intervention to demonstrate its full effects. Similarly, Miremberg et al [25] reported no significant differences in adverse pregnancy outcomes between groups. The inconsistency between these studies and our findings may be attributed to their populations’ significantly higher baseline BMI and relatively smaller sample sizes, which could have limited statistical power to detect meaningful differences.

In our study, the telemedicine-enhanced integrated management approach was significantly associated with decreased GWG and lower risk of EGWG, with exploratory analyses demonstrating a pronounced dose-response relationship between higher telemedicine-enhanced engagement levels and reduced EGWG rate. Similar findings were observed in 2 previous studies [26,27], although 1 contradictory study reported no significant EGWG differences between intervention and usual care groups [28], likely due to limited patient engagement (only 49.4% receiving app education and 68% maintaining weekly weight records). Given the established association between EGWG and increased LGA risk [29], we also observed lower LGA risk in the telemedicine-enhanced group, with the protective effect strengthening as engagement levels increased. While the standard care group exhibited higher LGA risk and a trend toward increased macrosomia, they paradoxically showed lower overall birth weights. This contradiction may be explained by their significantly higher preterm birth rate. Our observation of significant negative correlations between telemedicine-enhanced engagement levels and risks of both LGA and macrosomia in subgroup analyses further supports these findings. Although the TangMama app was introduced after GDM diagnosis, the exact timing of registration and intensity of subsequent app use within the postdiagnosis window varied among participants. Such variation in engagement may have contributed to differences in the observed associations. Nevertheless, the primary findings remained robust in sensitivity analyses addressing measured confounders and potential selection bias.

Mediation analyses showed that optimized GWG and glycemic control in the third trimester significantly mediated telemedicine-enhanced care’s effects on pregnancy outcomes. The substantial mediation effect of GWG, particularly for LGA risk reduction, supports the role of EGWG in fetal overgrowth [30], while the modest yet broad mediation effect of mean FPG in the third trimester underscores glycemic stability’s importance. These findings suggest that telemedicine-enhanced management may improve GDM outcomes through enhanced dietary and exercise supervision, optimizing both weight and glycemic control.

In this study, the telemedicine-enhanced group demonstrated significant reductions across multiple neonatal outcomes. While these findings are encouraging, they should be interpreted with caution. The integrated telemedicine-enhanced care model may strengthen overall disease management, leading to improved glycemic control and gestational weight management, which contribute to better pregnancy outcomes. Improvements in these key metabolic parameters may exert a disproportionate impact on certain adverse outcomes. Additionally, for outcomes with relatively low event rates, such as compromised Apgar scores at 1 and 5 minutes and respiratory distress, the observed ORs were based on a limited number of events and were accompanied by relatively wide CIs, indicating limited precision. As this is a real-world observational study, residual confounding and unmeasured differences in patient motivation or health care–seeking behavior may have also played a role in the magnitude of these associations.

Our findings may also partly reflect baseline differences in patient engagement and self-management behaviors due to self-selection. Women who voluntarily adopted the TangMama app may have possessed higher health literacy, stronger self-efficacy, and greater intrinsic motivation for self-management than nonadopters [31]. These characteristics are significantly associated with better glycemic control, dietary adherence, and gestational weight management in GDM [32,33]. Importantly, evidence from prior studies also suggests that telemedicine-based interventions can actively enhance patients’ disease knowledge, promote health-related behaviors, and improve diabetes self-efficacy [34,35]. Thus, the observed associations in our study likely reflect a combination of baseline differences in patient engagement and the potential for telemedicine to further enhance self-management capacity. Although direct measurement of these behavioral factors was not feasible in this study, our findings highlight the potential of integrated telemedicine approaches to support health literacy and empower women in GDM self-management.

GDM is highly prevalent, yet treatment efficacy is frequently constrained by the limited window for optimal glycemic control, suboptimal patient awareness, and the insufficient communication of conventional management approaches [35-37]. The number of smartphone mobile network subscriptions worldwide reached almost 7.3 billion in 2025, and is forecast to exceed 7.9 billion by 2028 [38]. The widespread adoption of smartphones creates a robust foundation for telemedicine implementation. Moreover, digital health integration demonstrates tremendous potential in addressing structural inequalities in health care resource distribution. China’s Healthy China 2030 identifies the advancement of digital health integration as one of the key medical optimization strategies, aiming to establish a nationwide telemedicine service network that enables the redistribution of high-quality medical resources to underserved regions [39,40]. As pivotal hubs for optimizing health care resource utilization, regional comprehensive medical centers can consolidate resources, foster network collaboration, leverage technological advancements, and enforce rigorous quality control to establish an integrated GDM management model spanning prevention, diagnosis, and follow-up care [41]. Through data stream integration between hospital systems and the digital app, the telemedicine-enhanced integrated management enables multidisciplinary expert teams to develop precise, individualized management protocols leveraging comprehensive hospital resources and efficient telemedicine-based tools. Additionally, formal hospital endorsement substantially enhances the platform’s perceived credibility, improving patient compliance and satisfaction while offering improved visit efficiency and cost-effectiveness. However, translating these advantages into real-world patient engagement remains challenging. In our study, of the 4621 women with GDM, only 1711 (37.0%) women engaged with the TangMama app, indicating substantial space for improvement of this strategy uptake.

### Limitations

Our study has several limitations. First, this is a nonrandomized observational study in which participation in the telemedicine-enhanced program was self-selected rather than randomly assigned. Although IPTW was applied to balance observed confounders between groups, unmeasured confounding factors such as baseline health literacy and socioeconomic status cannot be fully accounted for. Women who voluntarily adopted the TangMama app may inherently possess greater health awareness and adherence capacity, which could contribute to the observed improvements independently of the telemedicine intervention itself. Therefore, while our findings demonstrate significant associations between telemedicine-enhanced management and improved maternal-neonatal outcomes, causal inference should be made with caution. The single-center prospective cohort design may additionally limit the generalizability of our findings due to population characteristics and institutional practices. Future well-designed randomized controlled trials are warranted to confirm causality and establish the efficacy of this intervention. Furthermore, although the telemedicine-enhanced model demonstrated short-term clinical effectiveness, its long-term sustainability may be challenged by the need for continuous algorithm updating. Future research should focus on developing adaptive management systems capable of dynamic algorithm optimization.

### Conclusions

Our findings suggest that telemedicine-enhanced integrated management effectively improves maternal glycemic control and reduces adverse pregnancy outcomes among women with GDM. This innovative approach enhances patient-provider communication while delivering personalized care that increases patient engagement in self-management. The successful practice of telemedicine-enhanced integrated management carries broader implications for maternal-fetal health, potentially reducing long-term metabolic risks and advancing birth defect prevention strategies.

### Prior Presentation

This work was presented at the 59th EASD Annual Meeting of the European Association for the Study of Diabetes, October 2‐6, 2023.

## Supplementary material

10.2196/90487 Multimedia Appendix 1 Supplementary methods, outcome definitions, TangMama platform screenshots, study flowchart, sensitivity analyses, and mediation analyses..

## Abbreviations

aMD: adjusted mean difference
aOR: adjusted odds ratio
EGWG: excessive gestational weight gain
FPG: fasting plasma glucose
GDM: gestational diabetes mellitus
GWG: gestational weight gain
HbA_1c_: glycated hemoglobin
IPTW: inverse probability of treatment weighting
LBW: low birth weight
LGA: large-for-gestational age
OGTT: oral glucose tolerance test
OR: odds ratio
PS: propensity score
SGA: small-for-gestational age
SMBG: self-monitoring of blood glucose
