# Supplementary material for: Impact of Telemedicine-Enhanced Integrated Management of Gestational Diabetes on Pregnancy Outcomes and Glycemic Control: Real-World Study Using TangMama App
Source: J Med Internet Res. 2026 Jul 3;28:e90487. doi: 10.2196/90487 (PMC13331329; doi:10.2196/90487)
Supplement: Multimedia Appendix 1 [file jmir-v28-e90487-s001.docx]

Multimedia Appendix 1

**eMethods**

**Study Design:**

This study is a real-world, prospective cohort study conducted in a regional medical center. Women with GDM who attended Anhui Provincial Hospital between January 1, 2022, and October 1, 2024, were eligible for the study.

**Inclusion criterion:**

(1) aged 18-55 years;

(2) diagnosed with GDM using the WHO 2013 criteria: fasting plasma glucose ≥ 5.1mmol/L, or 60 min plasma glucose ≥ 10.0mmol/L, or 120 min plasma glucose ≥ 8.5mmol/L after a 75-g OGTT;

(3) delivered at Anhui Provincial Hospital;

(4) singleton pregnancy.

**Exclusion criteria:**

(1) pre-existing diabetes;

(2) multiple pregnancies;

(3) complications involving significant organ diseases or evident dysfunction of heart, liver, lung, or kidney;

(4) autoimmune diseases requiring prolonged use of glucocorticoids;

(5) eating disorders;

(6) history of weight-loss surgery.

**Table S1.** Definition of pregnancy outcomes.

| Outcomes |  | Definition |
| --- | --- | --- |
| Maternal outcomes | Gestational weight gain | Weight from preconception (preferable) (measured or self-reported) or within 3 months of conception, or if not available, at first pregnancy visit within first trimester, until the last measured weight during pregnancy (within 4 weeks of delivery). |
|  | Excessive gestational weight gain | gestational weight gain exceeding the recommended GWG range for each pre-pregnancy BMI category based on the 2009 Institute of Medicine guidelines |
|  | Caesarean section | Extraction of the foetus(es) through an abdominal incision in a woman |
|  | Normal vaginal delivery | Delivery through the vagina, without mechanical or pharmacological intervention. |
|  | Forceps delivery | Application of forceps to the foetal head. |
|  | Episiotomy | An incision made in the tissue between the vaginal opening and the anus during childbirth |
|  | Hypertensive disorders in pregnancy | Including gestational hypertension, chronic hypertension, preeclampsia, and eclampsia. |
|  | Preeclampsia | Definition *de novo* hypertension after gestational week 20 and the coexistence of one or more of the following new onset conditions:  1. Proteinuria (spot urine protein/creatinine ratio ≥ 30 mg/mmol (0.3 mg/mg) or ≥ 300 mg/day or at least 1 g/L (‘2+’) on dipstick testing)  2. Other maternal organ dysfunction:  • Renal insufficiency (creatinine ≥ 0.09 umol/L; 1.02 mg/dL)  • Liver involvement (elevated transaminases: at least twice upper limit of normal + right upper quadrant or epigastric abdominal pain)  • Neurological complications (examples include eclampsia, altered mental status, blindness, stoke, or more commonly hyperreflexia when accompanied by clonus, severe heaches when accompanied by hyperreflexia, persistent visual scotomata)  • Haematological complications (thrombocytopenia: platelet count below 150,000/dL, DIC, haemolysis)  3. Uteroplacental dysfunction Fetal growth restriction |
|  | Shoulder dystocia | Delivery that requires additional obstetric manoeuvres following failure of gentle downward traction on the foetal head to effect delivery of the shoulders including one or more of the following: suprapubic pressure, McRoberts manoeuvre, Wood’s screw manoeuvre or Rubin’s manoeuvres, delivery of the posterior arm, the Gaskin manoeuvre, the Zavanelli manoeuvre or intentional fracture of the clavicle or humerus. |
|  | Miscarriage | Spontaneous loss of a pregnancy before the 20th week of gestation. |
|  | Stillbirth | Delivery of a fetus showing no signs of life as indicated by the absence of breathing, heartbeats, pulsation of the umbilical cord, or definite movements of voluntary muscles at 20 weeks or greater of gestation. |
| Neonatal outcomes | Preterm birth | Delivery at < 37 weeks gestation |
|  | Large-for-gestational-age | Birth weight > 90th percentile for gestational age |
|  | Macrosomia | Birth weight ≥ 4000 g |
|  | Small-for-gestational-age | Birth weight < 10th percentile for gestational age |
|  | Low birth weight | Birth weight < 2500 g |
|  | Apgar scores | A measure of the health of a newborn infant done at 1 and 5 min. The newborn is given points (0, 1, 2) for heart rate, respiratory effort, muscle tone, response to stimulation and skin coloration. A score of 10 points indicates excellent health. |
|  | Neonatal unit admission | Admission to neonatal care unit or special care nursery for >24 hours during the  initial hospitalization after birth |
|  | Neonatal hypoglycemia | A plasma glucose value < 2.2 mmol/L |
|  | Neonatal hyperbilirubinemia | Need for phototherapy or exchange transfusion |
|  | Neonatal respiratory distress | Respiratory difficulties requiring any positive pressure ventilation ≧ 24 h that occurs beyond the first 10 min of the resuscitation period, and/or given surfactant within 72 h after birth. |
|  | Congenital heart defects | Malformations categorized under ICD 10 codes Q20 – Q28 |

**Table S2.** Sensitivity analysis of pregnancy outcomes using IPTW based on a propensity score model excluding insulin treatment.

|  | **Telemedicine-enhanced group (n=1709.1)** | **Standard care group (n=2911.0)** | **Crude OR or Mean difference (95% CI)** | ***p* value** | **Adjusted OR or Mean difference (95% CI)** | ***p* value** |
| --- | --- | --- | --- | --- | --- | --- |
| **Maternal Outcomes** | | | | | | |
| GWG, kg | 11.7 (5.3) | 13.2 (5.7) | -1.48 (-1.81 to -1.15) ^a^ | <0.001 | -1.48 (-1.80 to -1.16) ^a^ | <0.001 |
| EGWG % | 590.8 (34.6) | 1335.9 (45.9) | 0.62 (0.55 to 0.71) ^b^ | <0.001 | 0.61 (0.54 to 0.70) ^b^ | <0.001 |
| Gestational age at delivery, weeks | 38.95 (1.46) | 38.52 (2.14) | 0.43 (0.33 to 0.54) ^b^ | <0.001 | 0.43 (0.33 to 0.53) ^b^ | <0.001 |
| Mode of delivery | | | | | | |
| Cesarean section | 919.5 (53.8) | 1708.9 (58.7) | 0.82 (0.73 to 0.92) ^b^ | 0.001 | 0.81 (0.71 to 0.91) ^b^ | <0.001 |
| Normal vaginal delivery | 775.0 (45.3) | 1178.6 (40.5) | 1.22 (1.08 to 1.38) ^b^ | 0.001 | 1.24 (1.09 to 1.40) ^b^ | <0.001 |
| Forceps delivery | 14.6 (0.9) | 23.6 (0.8) | 1.06 (0.55 to 2.03) ^b^ | 0.870 | 1.10 (0.57 to 2.12) ^b^ | 0.778 |
| Episiotomy | 101.0 (5.9) | 184.2 (6.3) | 0.93 (0.73 to 1.19) ^b^ | 0.563 | 0.93 (0.72 to 1.19) ^b^ | 0.553 |
| Hypertensive disorders in pregnancy | 253.1 (14.8) | 537.4 (18.5) | 0.77 (0.65 to 0.90) ^b^ | 0.001 | 0.76 (0.65 to 0.91) ^b^ | 0.002 |
| Preeclampsia, | 80.6 (4.7) | 211.7 (7.3) | 0.63 (0.49 to 0.82) ^b^ | <0.001 | 0.64 (0.49 to 0.83) ^b^ | 0.001 |
| Shoulder dystocia | 1.8 (0.1) | 7.2 (0.2) | 0.43 (0.09 to 2.09) ^b^ | 0.298 | 0.43 (0.09 to 2.06) ^b^ | 0.290 |
| Pregnancy loss | 3.8 (0.2) | 29.3 (1.0) | 0.22 (0.08 to 0.62) ^b^ | 0.005 | 0.22 (0.08 to 0.63) ^b^ | 0.005 |
| Parameters of glycemic control | | | | | | |
| HbA_1c_ in the 3rd trimester, % | 5.57 (0.41) | 5.62 (0.43) | -0.06 (-0.08 to -0.03) ^c^ | <0.001 | -0.05 (-0.08 to -0.03) ^c^ | <0.001 |
| HbA_1c_ in the 3rd trimester < 6% | 1447.8 (84.7) | 2373.2 (81.5) | 1.26 (1.07 to 1.48) ^b^ | 0.006 | 1.23 (1.04 to 1.46) ^b^ | 0.016 |
| HbA_1c_ in the 3rd trimester < 5.6% | 944.7 (55.3) | 1442.8 (49.6) | 1.26 (1.11 to 1.42) ^b^ | <0.001 | 1.27 (1.12 to 1.44) ^b^ | <0.001 |
| Mean FPG in the 3rd trimester, mmol/L | 4.74 (0.56) | 4.81 (0.69) | -0.07 (-0.11 to -0.04) ^d^ | <0.001 | -0.07 (-0.10 to -0.03) ^d^ | <0.001 |
| **Neonatal Outcomes** | | | | | | |
| Preterm birth | 127.4 (7.5) | 419.7 (14.4) | 0.48 (0.39 to 0.59) ^b^ | <0.001 | 0.47 (0.38 to 0.58) ^b^ | <0.001 |
| LGA ^e^ | 257.0 (15.1) | 513.7 (17.8) | 0.82 (0.69 to 0.96) ^b^ | 0.016 | 0.82 (0.66 to 0.97) ^b^ | 0.020 |
| Macrosomia ^e^ | 137.4 (8.1) | 260.4 (9.0) | 0.88 (0.71 to 1.09) ^b^ | 0.256 | 0.72 (0.66 to 1.11) ^b^ | 0.306 |
| SGA ^e^ | 87.5 (5.1) | 177.3 (6.2) | 0.82 (0.63 to 1.07) ^b^ | 0.152 | 0.82 (0.63 to 1.08) ^b^ | 0.154 |
| LBW ^e^ | 61.6 (3.6) | 252.2 (8.8) | 0.39 (0.29 to 0.52) ^b^ | <0.001 | 0.39 (0.30 to 0.52) ^b^ | <0.001 |
| Birth weight, kg ^e^ | 3.31 (0.48) | 3.24 (0.59) | 0.07 (0.04 to 0.10) ^a^ | <0.001 | 0.07 (0.04 to 0.10) ^a^ | <0.001 |
| Birth length, cm ^e^ | 50.05 (1.93) | 49.58 (2.64) | 0.47 (0.34 to 0.61) ^a^ | <0.001 | 0.47 (0.34 to 0.61) ^a^ | <0.001 |
| Apgar score < 7 at 1 min ^e^ | 11.8 (0.7) | 48.1 (1.7) | 0.41 (0.22 to 0.79) ^b^ | 0.006 | 0.41 (0.22 to 0.77) ^b^ | 0.006 |
| Apgar score < 7 at 5 min ^e^ | 1.9 (0.1) | 18.9 (0.7) | 0.17 (0.04 to 0.71) ^b^ | 0.016 | 0.17 (0.04 to 0.71) ^b^ | 0.016 |
| Neonatal unit admission ^e^ | 685.3 (40.1) | 1323.8 (45.5) | 0.80 (0.71 to 0.91) ^b^ | <0.001 | 0.80 (0.71 to 0.90) ^b^ | <0.001 |
| Neonatal hypoglycemia ^e^ | 43.4 (2.5) | 113.0 (3.9) | 0.64 (0.45 to 0.92) ^b^ | 0.017 | 0.65 (0.45 to 0.93) ^b^ | 0.018 |
| Neonatal hyperbilirubinemia ^e^ | 200.4 (11.7) | 474.0 (16.3) | 0.68 (0.57 to 0.82) ^b^ | <0.001 | 0.68 (0.57 to 0.82) ^b^ | <0.001 |
| Neonatal respiratory distress ^e^ | 18.6 (1.1) | 93.8 (3.2) | 0.35 (0.20 to 0.55) ^b^ | <0.001 | 0.35 (0.20 to 0.55) ^b^ | <0.001 |
| Congenital heart defects ^e^ | 400.2 (23.4) | 836.7 (28.7) | 0.76 (0.66 to 0.87) ^b^ | <0.001 | 0.76 (0.66 to 0.87) ^b^ | <0.001 |
| Neonatal death ^e^ | 0.9 (0.1) | 3.9 (0.1) | 0.41 (0.05 to 3.64) ^b^ | 0.420 | 0.42 (0.05 to 3.82) ^b^ | 0.445 |

Abbreviations: IPTW, inverse probability of treatment weighting; OR, odds ratio; CI, confidence interval; HbA1c, glycated hemoglobin A1c; GWG, gestational weight gain; EGWG, excessive gestational weight gain; LGA, large-for-gestational-age; SGA, small-for-gestational-age; LBW, low birth weight.

Maternal age, education level, parity, pre-pregnancy BMI, and 1-hour post-load glucose on the OGTT were included in the propensity score model for IPTW.

^a^ The mean difference is estimated from a linear regression model; in the adjusted analysis, the adjusted mean difference was adjusted for maternal age, education level, parity, pre-pregnancy body mass index, 1-hour post-load glucose on oral glucose tolerance test, and insulin treatment.

^b^ The OR is estimated from a logistic regression model; in the adjusted analysis, the adjusted OR was adjusted for maternal age, education level, parity, pre-pregnancy body mass index, 1-hour post-load glucose on oral glucose tolerance test, and insulin treatment.

^c^ The OR is estimated from a logistic regression model; in the adjusted analysis, the adjusted OR was adjusted for maternal age, education level, parity, pre-pregnancy body mass index, 1-hour post-load glucose on oral glucose tolerance test, insulin treatment, and gestational age.

^d^ The mean difference is estimated from a linear regression model; in the adjusted analysis, the adjusted mean difference was adjusted for maternal age, education level, parity, pre-pregnancy body mass index, 1-hour post-load glucose on oral glucose tolerance test, insulin treatment, and gestational age.

^e^ 33.1 pregnancy losses were excluded from the analysis (3.8 in the Telemedicine-enhanced group, 29.3 in the Standard care group).

**Table S3.** Sensitivity analysis of women with gestational diabetes with propensity score matching.

|  | **Telemedicine-enhanced group (n=1695)** | **Standard care group (n=1695)** | **Crude OR or Mean difference (95% CI)** | ***p* value** | **Adjusted OR or Mean difference (95% CI)** | ***p* value** |
| --- | --- | --- | --- | --- | --- | --- |
| **Maternal Outcomes** | | | | | | |
| GWG, kg | 11.6 (5.3) | 13.2 (5.8) | -1.54 (-1.91 to -1.16) ^a^ | <0.001 | -1.53 (-1.90 to -1.16) ^a^ | <0.001 |
| EGWG % | 585 (34.5) | 759 (44.8) | 0.65 (0.57 to 0.75) ^b^ | <0.001 | 0.63 (0.55 to 0.73) ^b^ | <0.001 |
| Gestational age at delivery, weeks | 39.0 (1.5) | 38.5 (2.2) | 0.44 (0.32 to 0.57) ^b^ | <0.001 | 0.44 (0.32 to 0.56) ^b^ | <0.001 |
| Mode of delivery |  |  |  |  |  |  |
| Cesarean section | 910 (53.7) | 968 (57.1) | 0.87 (0.76 to 0.99) ^b^ | 0.045 | 0.86 (0.75 to 0.99) ^b^ | 0.036 |
| Normal vaginal delivery | 770 (45.4) | 710 (41.9) | 1.15 (1.01 to 1.32) ^b^ | 0.038 | 1.17 (1.01 to 1.34) ^b^ | 0.031 |
| Forceps delivery | 15 (0.9) | 17 (1.0) | 0.88 (0.43 to 1.78) ^b^ | 0.723 | 0.93 (0.45 to 1.88) ^b^ | 0.836 |
| Episiotomy | 106 (6.3) | 118 (7.0) | 0.89 (0.68 to 1.17) ^b^ | 0.407 | 0.89 (0.67 to 1.18) ^b^ | 0.418 |
| Hypertensive disorders in pregnancy | 258 (15.2) | 319 (18.8) | 0.77 (0.65 to 0.93) ^b^ | 0.005 | 0.76 (0.63 to 0.92) ^b^ | 0.004 |
| Preeclampsia, | 81 (4.8) | 127 (7.5) | 0.62 (0.46 to 0.82) ^b^ | 0.001 | 0.62 (0.46 to 0.83) ^b^ | 0.001 |
| Shoulder dystocia | 2 (0.1) | 6 (0.4) | 0.33 (0.05 to 1.45) ^b^ | 0.178 | 0.32 (0.05 to 1.39) ^b^ | 0.163 |
| Pregnancy loss | 4 (0.2) | 14 (0.8) | 0.28 (0.08 to 0.79) ^b^ | 0.027 | 0.28 (0.08 to 0.80) ^b^ | 0.027 |
| Parameters of glycemic control | | | | | | |
| HbA_1c_ in the 3rd trimester, % | 5.57 (0.41) | 5.61 (0.42) | -0.04 (-0.07 to -0.01) ^c^ | 0.009 | -0.03 (-0.06 to -0.01) ^c^ | 0.015 |
| HbA_1c_ in the 3rd trimester < 6% | 1435 (84.7) | 1400 (82.6) | 1.16 (0.97 to 1.40) ^b^ | 0.104 | 1.13 (0.93 to 1.36) ^b^ | 0.212 |
| HbA_1c_ in the 3rd trimester < 5.6% | 931 (54.9) | 867 (51.2) | 1.16 (1.02 to 1.33) ^b^ | 0.028 | 1.16 (1.01 to 1.33) ^b^ | 0.035 |
| Mean FPG in the 3rd trimester, mmol/L | 4.74 (0.58) | 4.77 (0.66) | -0.06 (-0.10 to -0.02) ^d^ | 0.002 | -0.06 (-0.10 to -0.02) ^d^ | 0.004 |
| **Neonatal Outcomes** | | | | | | |
| Preterm birth | 126 (7.4) | 245 (14.5) | 0.48 (0.38 to 0.59) ^b^ | <0.001 | 0.47 (0.37 to 0.59) ^b^ | <0.001 |
| LGA ^e^ | 258 (15.3) | 306 (18.2) | 0.81 (0.67 to 0.97) ^b^ | 0.022 | 0.81 (0.67 to 0.97) ^b^ | 0.024 |
| Macrosomia ^e^ | 138 (8.2) | 161 (9.6) | 0.84 (0.66 to 1.06) ^b^ | 0.148 | 0.84 (0.66 to 1.06) ^b^ | 0.148 |
| SGA ^e^ | 86 (5.1) | 98 (5.8) | 0.87 (0.64 to 1.17) ^b^ | 0.342 | 0.87 (0.64 to 1.17) ^b^ | 0.355 |
| LBW ^e^ | 61 (3.6) | 137 (8.1) | 0.42 (0.31 to 0.57) ^b^ | <0.001 | 0.42 (0.30 to 0.57) ^b^ | <0.001 |
| Birth weight, kg ^e^ | 3.31 (0.48) | 3.24 (0.58) | 0.07 (0.03 to 0.10) ^a^ | <0.001 | 0.07 (0.03 to 0.10) ^a^ | <0.001 |
| Birth length, cm ^e^ | 50.1 (1.9) | 49.6 (2.6) | 0.48 (0.33 to 0.64) ^a^ | <0.001 | 0.48 (0.33 to 0.64) ^a^ | <0.001 |
| Apgar score < 7 at 1 min ^e^ | 12 (0.7) | 20 (1.2) | 0.59 (0.28 to 1.20) ^b^ | 0.155 | 0.58 (0.27 to 1.17) ^b^ | 0.137 |
| Apgar score < 7 at 5 min ^e^ | 2 (0.1) | 8 (0.5) | 0.25 (0.04 to 0.99) ^b^ | 0.078 | 0.24 (0.04 to 0.97) ^b^ | 0.073 |
| Neonatal unit admission ^e^ | 688 (40.6) | 778 (45.9) | 0.81 (0.70 to 0.90) ^b^ | 0.002 | 0.81 (0.70 to 0.90) ^b^ | 0.002 |
| Neonatal hypoglycemia ^e^ | 43 (2.5) | 67 (4.0) | 0.63 (0.43 to 0.93) ^b^ | 0.021 | 0.64 (0.43 to 0.95) ^b^ | 0.026 |
| Neonatal hyperbilirubinemia ^e^ | 202 (11.9) | 271 (16.0) | 0.71 (0.58 to 0.86) ^b^ | <0.001 | 0.72 (0.59 to 0.87) ^b^ | <0.001 |
| Neonatal respiratory distress ^e^ | 18 (1.1) | 45 (2.7) | 0.39 (0.22 to 0.67) ^b^ | <0.001 | 0.39 (0.22 to 0.66) ^b^ | <0.001 |
| Congenital heart defects ^e^ | 400 (23.6) | 507 (29.9) | 0.72 (0.62 to 0.84) ^b^ | <0.001 | 0.73 (0.62 to 0.85) ^b^ | <0.001 |
| Neonatal death ^e^ | 1 (0.1) | 0 (0.0) | NA ^f^ | NA ^f^ | NA ^f^ | NA ^f^ |

Abbreviations: OR, odds ratio; CI, confidence interval; HbA_1c_, glycated hemoglobin A_1c_; GWG, gestational weight gain; EGWG, excessive gestational weight gain; LGA, large-for-gestational-age; SGA, small-for-gestational-age; LBW, low birth weight.

^a^ The mean difference is estimated from a linear regression model; in the adjusted analysis, the adjusted mean difference was adjusted for maternal age, education level, parity, pre-pregnancy body mass index, 1-hour post-load glucose on oral glucose tolerance test, and insulin treatment.

^b^ The OR is estimated from a logistic regression model; in the adjusted analysis, the adjusted OR was adjusted for maternal age, education level, parity, pre-pregnancy body mass index, 1-hour post-load glucose on oral glucose tolerance test, and insulin treatment.

^c^ The OR is estimated from a logistic regression model; in the adjusted analysis, the adjusted OR was adjusted for maternal age, education level, parity, pre-pregnancy body mass index, 1-hour post-load glucose on oral glucose tolerance test, insulin treatment, and gestational age.

^d^ The mean difference is estimated from a linear regression model; in the adjusted analysis, the adjusted mean difference was adjusted for maternal age, education level, parity, pre-pregnancy body mass index, 1-hour post-load glucose on oral glucose tolerance test, insulin treatment, and gestational age.

^e^ 18 pregnancy losses were excluded from the analysis (4 in the Telemedicine-enhanced group, 14 in the Standard care group).

^f^ The effect size was judged to be clinically not interpretable.

**Table S4.** Sensitivity analysis in nulliparous women with gestational diabetes.

|  | **Telemedicine-enhanced group (n=1080)** | **Standard care group (n=1640)** | **Crude OR or Mean difference (95% CI)** | ***p* value** | **Adjusted OR or Mean difference (95% CI)** | ***p* value** |
| --- | --- | --- | --- | --- | --- | --- |
| **Maternal Outcomes** | | | | | | |
| GWG, kg | 11.7 (5.4) | 13.2 (5.7) | -1.44 (-1.87 to -1.02) ^a^ | <0.001 | -1.38 (-1.80 to -0.95) ^a^ | <0.001 |
| EGWG % | 370 (34.3) | 721 (44.0) | 0.66 (0.57 to 0.78) ^b^ | <0.001 | 0.64 (0.54 to 0.75) ^b^ | <0.001 |
| Gestational age at delivery, weeks | 39.2 (1.4) | 38.8 (2.1) | 0.33 (0.19 to 0.47) ^b^ | <0.001 | 0.33 (0.19 to 0.47) ^b^ | <0.001 |
| Mode of delivery |  |  |  |  |  |  |
| Cesarean section | 519 (48.1) | 862 (52.6) | 0.83 (0.72 to 0.97) ^b^ | 0.022 | 0.81 (0.69 to 0.95) ^b^ | 0.010 |
| Normal vaginal delivery | 548 (50.7) | 757 (46.2) | 1.20 (1.03 to 1.40) ^b^ | 0.019 | 1.23 (1.05 to 1.44) ^b^ | 0.010 |
| Forceps delivery | 13 (1.2) | 21 (1.3) | 0.94 (0.46 to 1.86) ^b^ | 0.860 | 1.03 (0.50 to 2.05) ^b^ | 0.934 |
| Episiotomy | 105 (9.7) | 169 (10.3) | 0.94 (0.72 to 1.21) ^b^ | 0.621 | 0.95 (0.73 to 1.23) ^b^ | 0.669 |
| Hypertensive disorders in pregnancy | 177 (16.4) | 308 (18.8) | 0.85 (0.69 to 1.04) ^b^ | 0.111 | 0.81 (0.65 to 1.00) ^b^ | 0.052 |
| Preeclampsia, | 55 (5.1) | 123 (7.5) | 0.66 (0.47 to 0.91) ^b^ | 0.014 | 0.64 (0.46 to 0.89) ^b^ | 0.009 |
| Shoulder dystocia | 2 (0.2) | 5 (0.3) | 0.61 (0.09 to 2.82) ^b^ | 0.551 | 0.58 (0.08 to 2.71) ^b^ | 0.520 |
| Pregnancy loss | 3 (0.3) | 12 (0.7) | 0.34 (0.09 to 1.19) ^b^ | 0.132 | 0.37 (0.08 to 1.17) ^b^ | 0.126 |
| Parameters of glycemic control |  |  |  |  |  |  |
| HbA_1c_ in the 3rd trimester, % | 5.57 (0.41) | 5.61 (0.42) | -0.04 (-0.07 to -0.01) ^c^ | 0.026 | -0.04 (-0.07 to -0.01) ^c^ | 0.005 |
| HbA_1c_ in the 3rd trimester < 6% | 919 (85.1) | 1374 (83.8) | 1.11 (0.89 to 1.37) ^b^ | 0.375 | 1.14 (0.91 to 1.42) ^b^ | 0.260 |
| HbA_1c_ in the 3rd trimester < 5.6% | 592 (54.8) | 821 (50.1) | 1.21 (1.04 to 1.41) ^b^ | 0.015 | 1.23 (1.09 to 1.50) ^b^ | 0.003 |
| Mean FPG in the 3rd trimester, mmol/L | 4.74 (0.58) | 4.77 (0.66) | -0.03 (-0.08 to 0.01) ^d^ | 0.162 | -0.04 (-0.08 to 0.01) ^d^ | 0.115 |
| **Neonatal Outcomes** | | | | | | |
| Preterm birth | 65 (6.0) | 186 (11.3) | 0.50 (0.37 to 0.67) ^b^ | <0.001 | 0.49 (0.36 to 0.66) ^b^ | <0.001 |
| LGA ^e^ | 148 (13.7) | 216 (13.3) | 1.04 (0.83 to 1.30) ^b^ | 0.724 | 1.02 (0.81 to 1.28) ^b^ | 0.884 |
| Macrosomia ^e^ | 86 (8.0) | 143 (8.8) | 0.90 (0.68 to 1.19) ^b^ | 0.465 | 0.87 (0.65 to 1.15) ^b^ | 0.339 |
| SGA ^e^ | 67 (6.2) | 115 (7.1) | 0.87 (0.64 to 1.19) ^b^ | 0.392 | 0.88 (0.64 to 1.20) ^b^ | 0.427 |
| LBW ^e^ | 38 (3.5) | 132 (8.1) | 0.41 (0.28 to 0.59) ^b^ | <0.001 | 0.41 (0.28 to 0.59) ^b^ | <0.001 |
| Birth weight, kg ^e^ | 3.31 (0.48) | 3.24 (0.58) | 0.07 (0.03 to 0.11) ^a^ | 0.001 | 0.06 (0.02 to 0.10) ^a^ | 0.003 |
| Birth length, cm ^e^ | 50.1 (1.9) | 49.6 (2.5) | 0.41 (0.24 to 0.59) ^a^ | <0.001 | 0.40 (0.23 to 0.58) ^a^ | <0.001 |
| Apgar score < 7 at 1 min ^e^ | 8 (0.7) | 27 (1.7) | 0.44 (0.19 to 0.94) ^b^ | 0.045 | 0.44 (0.19 to 0.93) ^b^ | 0.042 |
| Apgar score < 7 at 5 min ^e^ | 2 (0.2) | 10 (0.6) | 0.30 (0.05 to 1.14) ^b^ | 0.122 | 0.30 (0.05 to 1.16) ^b^ | 0.126 |
| Neonatal unit admission ^e^ | 486 (45.0) | 772 (47.1) | 0.92 (0.79 to 1.07) ^b^ | 0.289 | 0.91 (0.78 to 1.06) ^b^ | 0.242 |
| Neonatal hypoglycemia ^e^ | 22 (2.0) | 47 (2.9) | 0.70 (0.41 to 1.16) ^b^ | 0.181 | 0.68 (0.40 to 1.13) ^b^ | 0.145 |
| Neonatal hyperbilirubinemia ^e^ | 142 (13.1) | 276 (16.8) | 0.75 (0.60 to 0.93) ^b^ | 0.009 | 0.75 (0.60 to 0.93) ^b^ | 0.009 |
| Neonatal respiratory distress ^e^ | 5 (0.5) | 46 (2.8) | 0.16 (0.06 to 0.37) ^b^ | <0.001 | 0.16 (0.05 to 0.36) ^b^ | <0.001 |
| Congenital heart defects ^e^ | 289 (26.8) | 486 (29.6) | 0.87 (0.73 to 1.03) ^b^ | 0.104 | 0.86 (0.72 to 1.02) ^b^ | 0.085 |
| Neonatal death ^e^ | 1 (0.1) | 1 (0.1) | 1.52 (0.06 to 38.44) ^b^ | 0.768 | 1.37 (0.05 to 34.99) ^b^ | 0.825 |

Abbreviations: OR, odds ratio; CI, confidence interval; HbA_1c_, glycated hemoglobin A_1c_; GWG, gestational weight gain; EGWG, excessive gestational weight gain; LGA, large-for-gestational-age; SGA, small-for-gestational-age; LBW, low birth weight.

^a^ The mean difference is estimated from a linear regression model; in the adjusted analysis, the adjusted mean difference was adjusted for maternal age, education level, pre-pregnancy body mass index, 1-hour post-load glucose on oral glucose tolerance test, and insulin treatment.

^b^ The OR is estimated from a logistic regression model; in the adjusted analysis, the adjusted OR was adjusted for maternal age, education level, pre-pregnancy body mass index, 1-hour post-load glucose on oral glucose tolerance test, and insulin treatment.

^c^ The OR is estimated from a logistic regression model; in the adjusted analysis, the adjusted OR was adjusted for maternal age, education level, parity, pre-pregnancy body mass index, 1-hour post-load glucose on oral glucose tolerance test, insulin treatment, and gestational age.

^d^ The mean difference is estimated from a linear regression model; in the adjusted analysis, the adjusted mean difference was adjusted for maternal age, education level, parity, pre-pregnancy body mass index, 1-hour post-load glucose on oral glucose tolerance test, insulin treatment, and gestational age.

^e^ 15 pregnancy losses were excluded from the analysis (3 in the Telemedicine-enhanced group, 12 in the Standard care group).

**Table S5.** Sensitivity analysis excluding participants who registered with the TangMama app after 28 weeks of gestation.

|  | **Telemedicine-enhanced group (n=1121)** | **Standard care group (n=2910)** | **Crude OR or Mean difference (95% CI)** | ***p* value** | **Adjusted OR or Mean difference (95% CI)** | ***p* value** |
| --- | --- | --- | --- | --- | --- | --- |
| **Maternal Outcomes** | | | | | | |
| GWG, kg | 11.2 (5.2) | 13.2 (5.7) | -2.04 (-2.43 to -1.66) ^a^ | <0.001 | -1.92 (-2.30 to -1.54) ^a^ | <0.001 |
| EGWG % | 342 (30.5) | 1338 (46.0) | 0.52 (0.45 to 0.60) ^b^ | <0.001 | 0.51 (0.44 to 0.60) ^b^ | <0.001 |
| Gestational age at delivery, weeks | 38.9 (1.5) | 38.5 (2.1) | 0.342 (0.28 to 0.55) ^b^ | <0.001 | 0.37 (0.23 to 0.50) ^b^ | <0.001 |
| Mode of delivery |  |  |  |  |  |  |
| Cesarean section | 592 (52.8) | 1710 (58.8) | 0.79 (0.68 to 0.90) ^b^ | <0.001 | 0.79 (0.68 to 0.91) ^b^ | 0.001 |
| Normal vaginal delivery | 520 (46.4) | 1177 (40.4) | 1.27 (1.11 to 1.46) ^b^ | <0.001 | 1.27 (1.10 to 1.47) ^b^ | <0.001 |
| Forceps delivery | 9 (0.8) | 23 (0.8) | 1.02 (0.44 to 2.13) ^b^ | 0.968 | 0.96 (0.42 to 2.04) ^b^ | 0.923 |
| Episiotomy | 75 (6.7) | 178 (6.1) | 1.10 (0.83 to 1.45) ^b^ | 0.501 | 0.97 (0.72 to 1.28) ^b^ | 0.820 |
| Hypertensive disorders in pregnancy | 173 (15.4) | 528 (18.1) | 0.82 (0.68 to 0.99) ^b^ | 0.042 | 0.77 (0.63 to 0.94) ^b^ | 0.009 |
| Preeclampsia, | 56 (5.0) | 208 (7.1) | 0.68 (0.50 to 0.92) ^b^ | 0.014 | 0.65 (0.47 to 0.89) ^b^ | 0.007 |
| Shoulder dystocia | 2 (0.2) | 7 (0.2) | 0.74 (0.11 to 3.07) ^b^ | 0.709 | 0.68 (0.10 to 2.84) ^b^ | 0.631 |
| Pregnancy loss | 4 (0.4) | 30 (1.0) | 0.34 (0.10 to 0.87) ^b^ | 0.045 | 0.37 (0.11 to 0.96) ^b^ | 0.066 |
| Parameters of glycemic control | | | | | | |
| HbA_1c_ in the 3rd trimester, % | 5.55 (0.42) | 5.62 (0.43) | -0.07 (-0.10 to -0.04) ^c^ | <0.001 | -0.07 (-0.10 to -0.04) ^c^ | <0.001 |
| HbA_1c_ in the 3rd trimester < 6% | 959 (85.5) | 2378 (81.7) | 1.32 (1.10 to 1.61) ^b^ | 0.004 | 1.34 (1.10 to 1.64) ^b^ | 0.004 |
| HbA_1c_ in the 3rd trimester < 5.6% | 638 (56.9) | 1453 (49.9) | 1.32 (1.15 to 1.52) ^b^ | <0.001 | 1.40 (1.21 to 1.62) ^b^ | <0.001 |
| Mean FPG in the 3rd trimester, mmol/L | 4.73 (0.57) | 4.81 (0.69) | -0.08 (-0.13 to -0.04) ^d^ | <0.001 | -0.08 (-0.12 to -0.03) ^d^ | <0.001 |
| **Neonatal Outcomes** | | | | | | |
| Preterm birth | 89 (7.9) | 424 (14.6) | 0.51 (0.40 to 0.64) ^b^ | <0.001 | 0.52 (0.41 to 0.66) ^b^ | <0.001 |
| LGA ^e^ | 161 (14.4) | 519 (18.0) | 0.77 (0.63 to 0.93) ^b^ | 0.007 | 0.79 (0.65 to 0.97) ^b^ | 0.022 |
| Macrosomia ^e^ | 81 (7.3) | 259 (9.0) | 0.79 (0.61 to 1.02) ^b^ | 0.077 | 0.80 (0.61 to 1.03) ^b^ | 0.084 |
| SGA ^e^ | 53 (4.7) | 176 (6.1) | 0.77 (0.55 to 1.04) ^b^ | 0.096 | 0.74 (0.54 to 1.01) ^b^ | 0.066 |
| LBW ^e^ | 41 (3.7) | 252 (8.8) | 0.40 (0.28 to 0.55) ^b^ | <0.001 | 0.40 (0.28 to 0.55) ^b^ | <0.001 |
| Birth weight, kg ^e^ | 3.29 (0.48) | 3.24 (0.59) | 0.05 (0.01 to 0.08) ^a^ | 0.021 | 0.05 (0.01 to 0.08) ^a^ | 0.019 |
| Birth length, cm ^e^ | 49.98 (1.93) | 49.57 (2.64) | 0.41 (0.24 to 0.58) ^a^ | <0.001 | 0.41 (0.23 to 0.58) ^a^ | <0.001 |
| Apgar score < 7 at 1 min ^e^ | 10 (0.9) | 48 (1.7) | 0.53 (0.25 to 1.01) ^b^ | 0.072 | 0.53 (0.25 to 1.01) ^b^ | 0.071 |
| Apgar score < 7 at 5 min ^e^ | 2 (0.2) | 19 (0.7) | 0.27 (0.04 to 0.93) ^b^ | 0.079 | 0.28 (0.04 to 0.96) ^b^ | 0.085 |
| Neonatal unit admission ^e^ | 442 (39.4) | 1315 (45.2) | 0.79 (0.69 to 0.91) ^b^ | <0.001 | 0.76 (0.65 to 0.87) ^b^ | <0.001 |
| Neonatal hypoglycemia ^e^ | 27 (2.4) | 113 (3.9) | 0.61 (0.39 to 0.92) ^b^ | 0.023 | 0.62 (0.40 to 0.94) ^b^ | 0.031 |
| Neonatal hyperbilirubinemia ^e^ | 134 (12.0) | 473 (16.3) | 0.70 (0.57 to 0.86) ^b^ | <0.001 | 0.70 (0.56 to 0.85) ^b^ | <0.001 |
| Neonatal respiratory distress ^e^ | 13 (1.2) | 94 (3.2) | 0.35 (0.19 to 0.61) ^b^ | <0.001 | 0.36 (0.19 to 0.62) ^b^ | <0.001 |
| Congenital heart defects ^e^ | 253 (22.6) | 830 (28.5) | 0.73 (0.62 to 0.86) ^b^ | <0.001 | 0.71 (0.60 to 0.83) ^b^ | <0.001 |
| Neonatal death ^e^ | 0 (0.0) | 4 (0.1) | NA ^f^ | NA ^f^ | NA ^f^ | NA ^f^ |

Abbreviations: OR, odds ratio; CI, confidence interval; HbA_1c_, glycated hemoglobin A_1c_; GWG, gestational weight gain; EGWG, excessive gestational weight gain; LGA, large-for-gestational-age; SGA, small-for-gestational-age; LBW, low birth weight.

^a^ The mean difference is estimated from a linear regression model; in the adjusted analysis, the adjusted mean difference was adjusted for maternal age, education level, parity, pre-pregnancy body mass index, 1-hour post-load glucose on oral glucose tolerance test, and insulin treatment.

^b^ The OR is estimated from a logistic regression model; in the adjusted analysis, the adjusted OR was adjusted for maternal age, education level, parity, pre-pregnancy body mass index, 1-hour post-load glucose on oral glucose tolerance test, and insulin treatment.

^c^ The OR is estimated from a logistic regression model; in the adjusted analysis, the adjusted OR was adjusted for maternal age, education level, parity, pre-pregnancy body mass index, 1-hour post-load glucose on oral glucose tolerance test, insulin treatment, and gestational age.

^d^ The mean difference is estimated from a linear regression model; in the adjusted analysis, the adjusted mean difference was adjusted for maternal age, education level, parity, pre-pregnancy body mass index, 1-hour post-load glucose on oral glucose tolerance test, insulin treatment, and gestational age.

^e^ 34 pregnancy losses were excluded from the analysis (4 in the Telemedicine-enhanced group, 30 in the Standard care group).

^f^ The effect size was judged to be clinically not interpretable.

**Figure S1.** Screenshots of key modules of TangMama (user terminal).


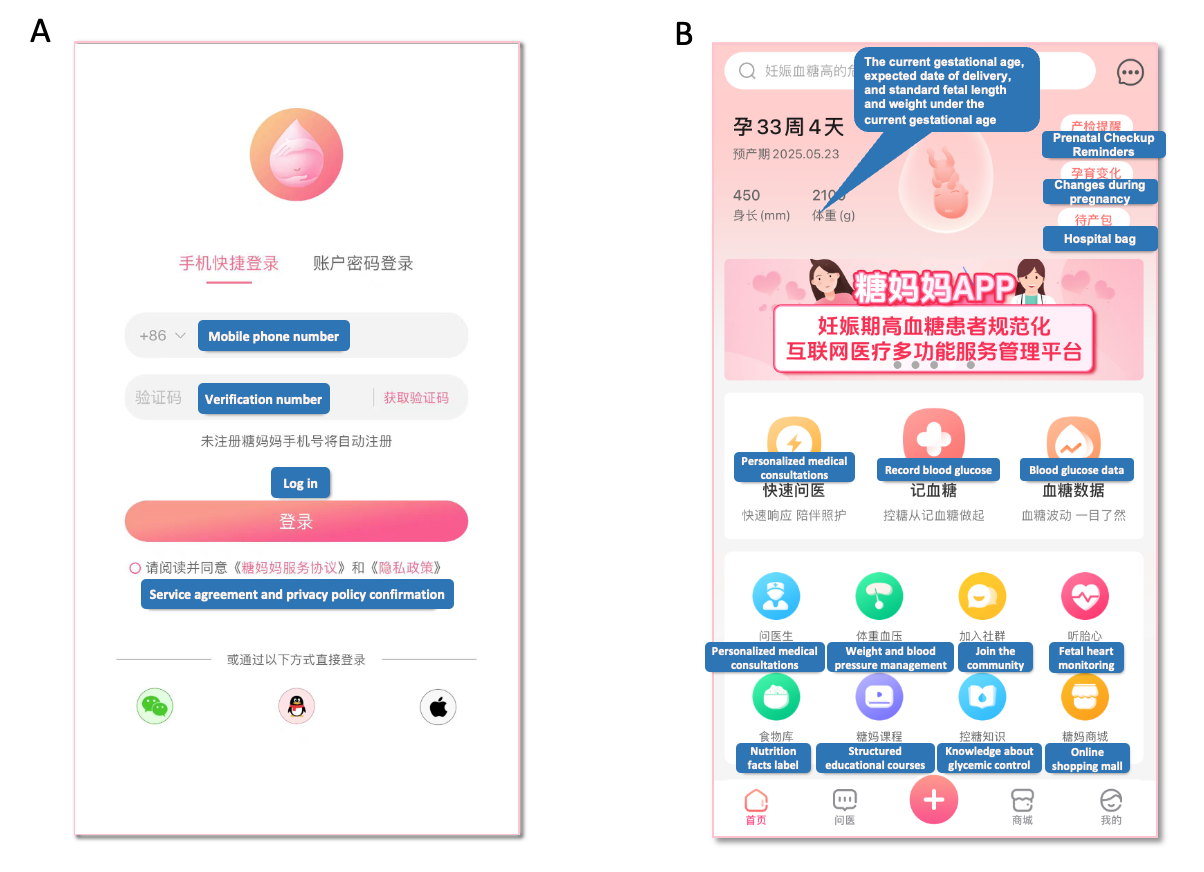


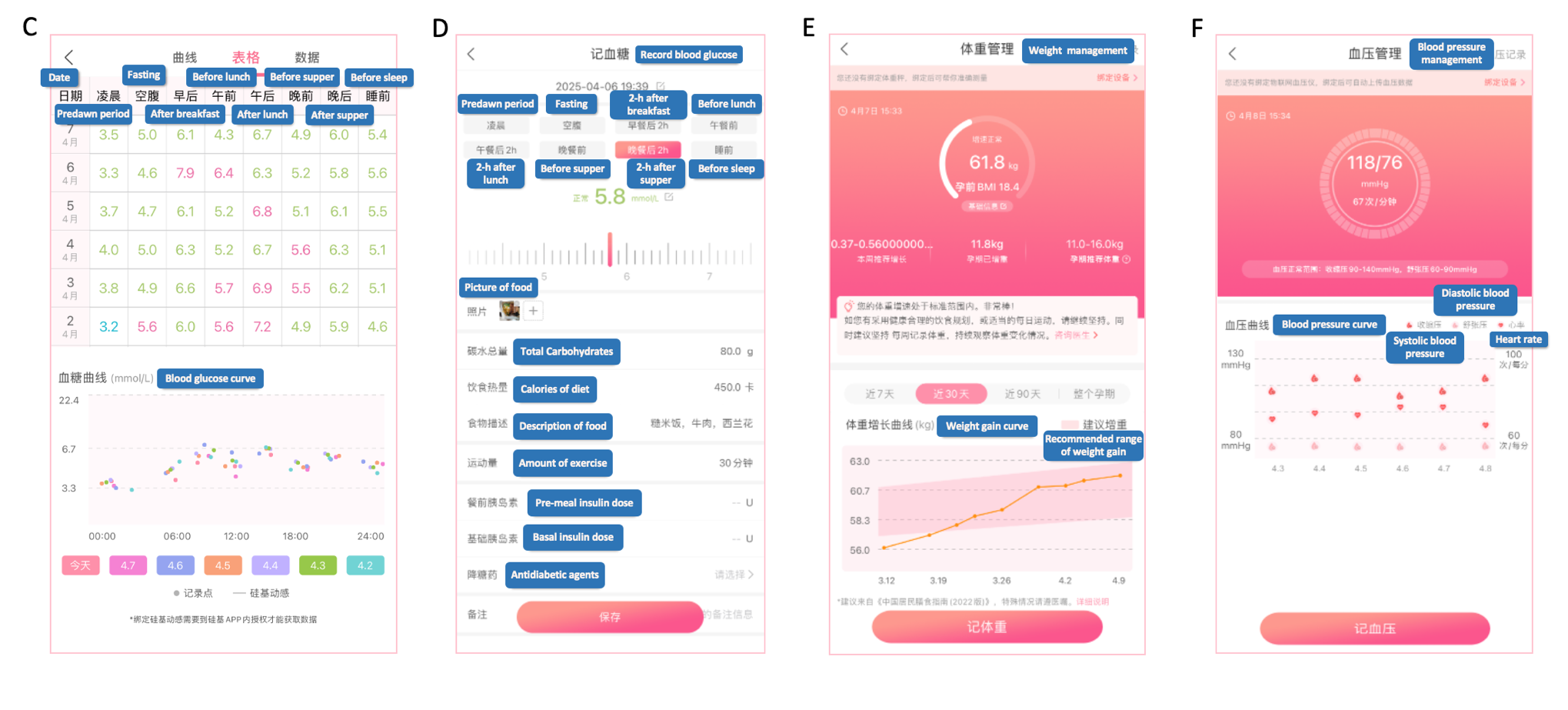


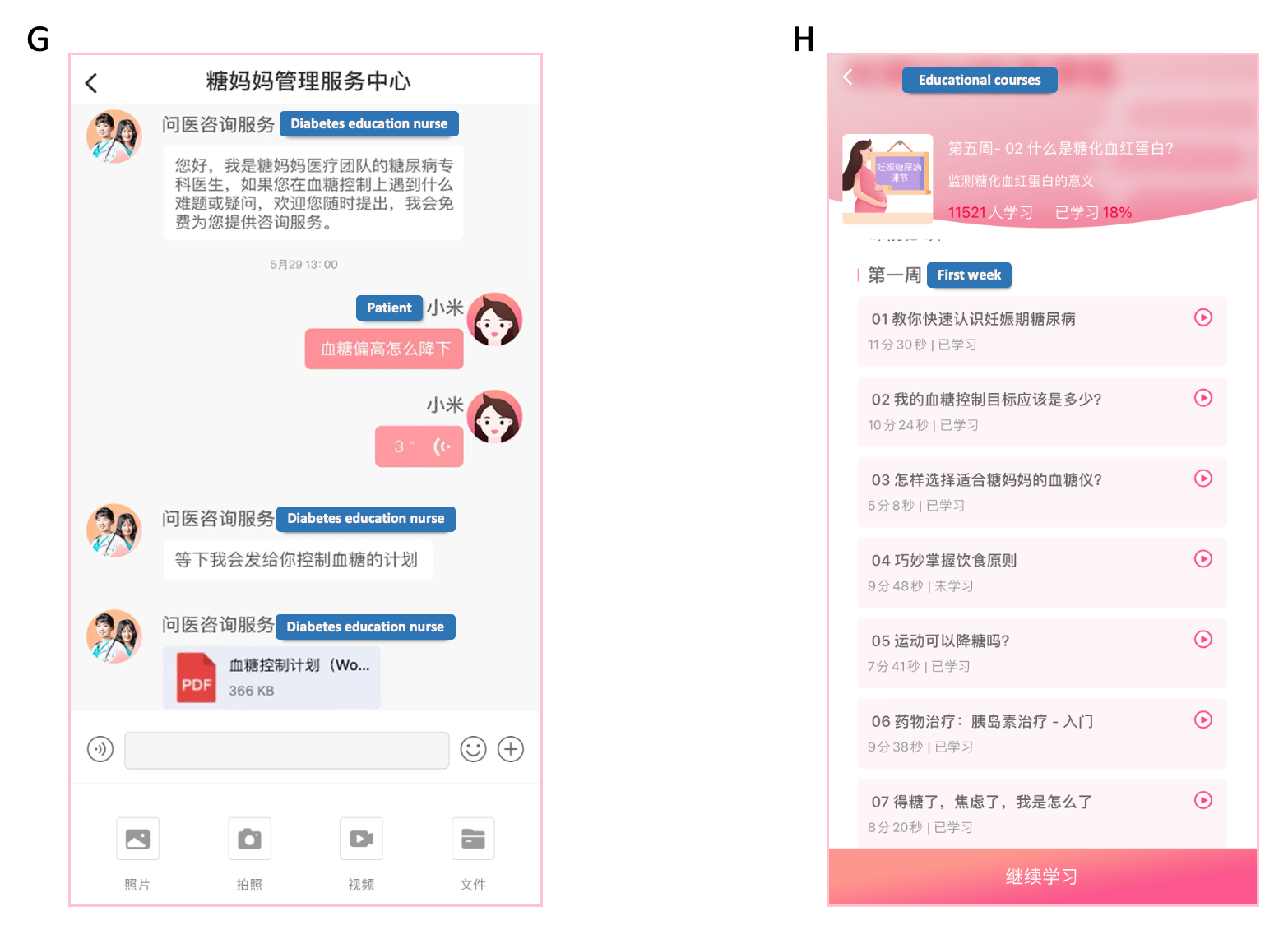


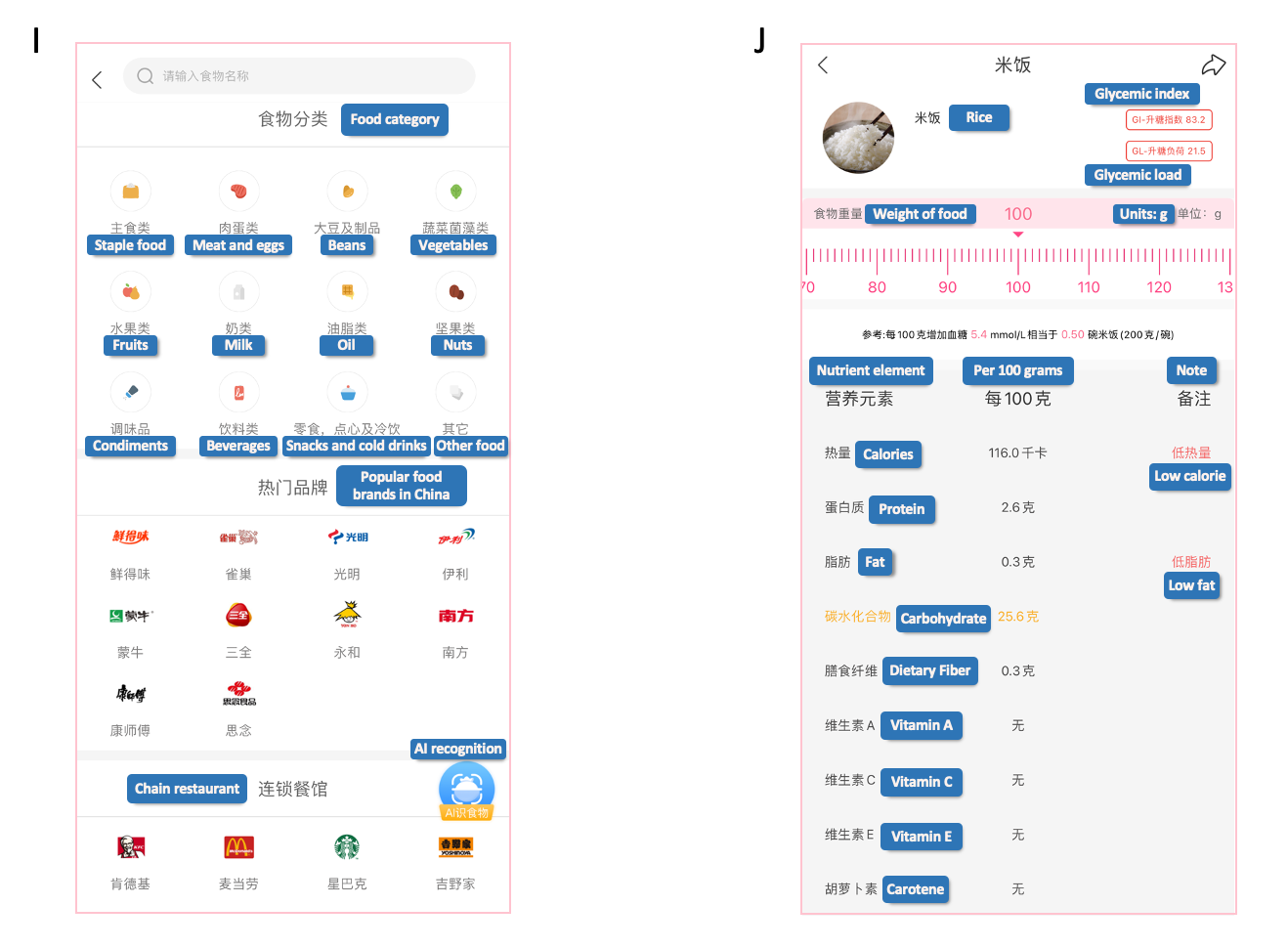


A-B. The login page and home page of the user terminal of the TangMama app.

C-F. Patient monitoring. Patients can input various health metrics including blood glucose, body weight, blood pressure, HbA1c, dietary intake, and medication manually or automatically uploaded through 5G-enabled smart devices. TangMama reminds patients of daily blood glucose levels based on the color change (green: the glucose level is stable and within the ideal range; pink: severe hyperglycemia; blue: severe hypoglycemia). When patients’ blood glucose levels are constantly out of range, the healthcare providers will offer them necessary guidance in time.

G. Personalized medicine support. Patients can contact the TangMama care team one-to-one via the in-app chat dashboard to receive timely advice on glycemic control, physical exercise, medical nutrition, and emotional support and obtain personalized treatment plans if necessary.

H. Structured educational module. Endocrinology and obstetrics experts have designed 59 high-quality educational courses tailored to the characteristics of different pregnancy stages, which cover a wide range of topics including self-monitoring of blood glucose, weight management, medication treatment, nutrition, and emotional management.

I-J. Dietary panel. Patients are able to quantify total calories and carbohydrates during a meal by selecting food items in grams in the dietary panel. This can also be achieved by using the AI Recognition function to take photos for precise food identification.


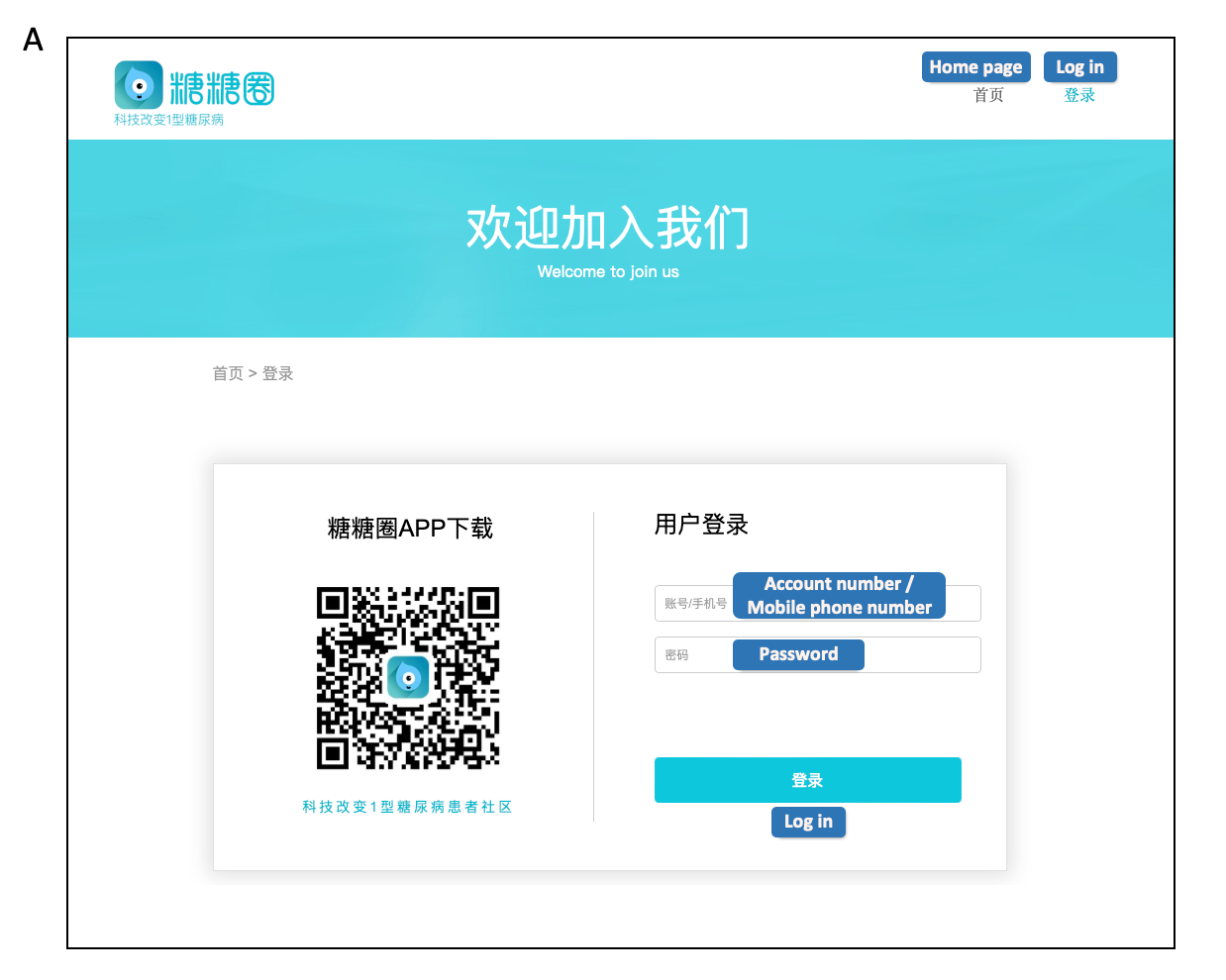
**Figure S2.** Screenshots of the interface of TangMama backend management system platform.


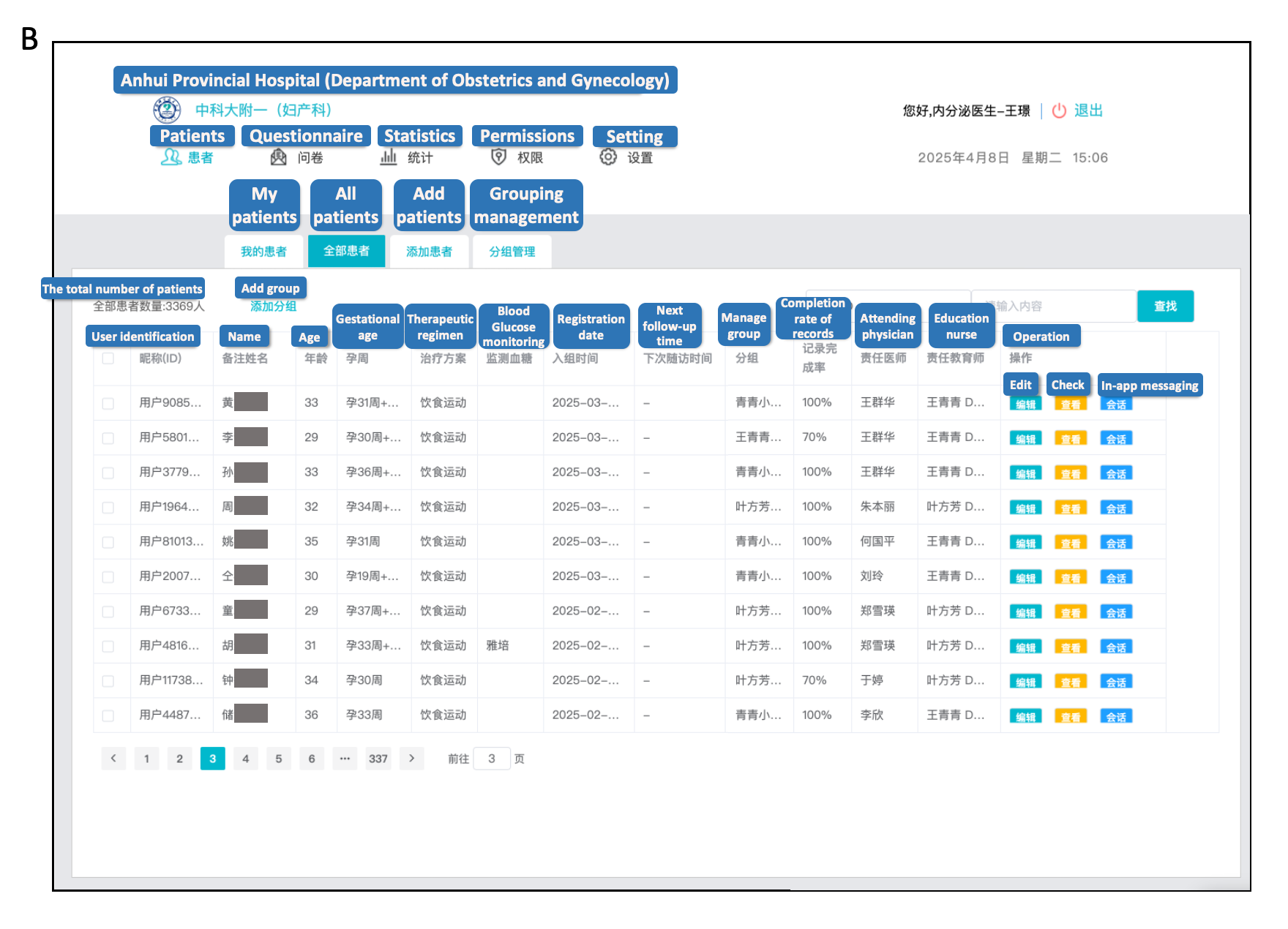


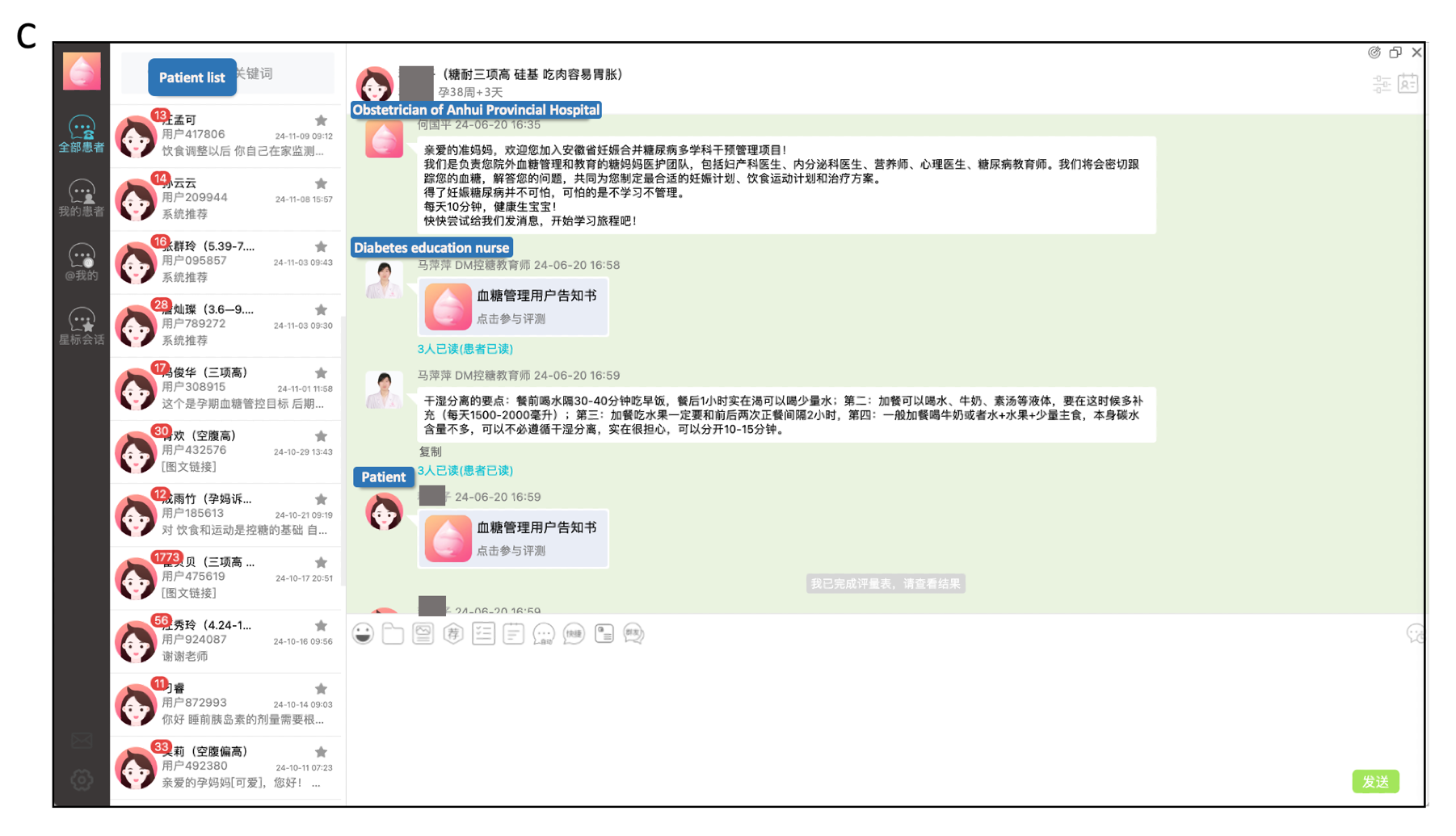
A. Login page of TangMama backend management system platform.

B. List of registered patients for TangMama.

C. Three-way communication portal. Patients, doctors, and diabetes education nurses can communicate in the same chat window.

**Figure S3.** The flowchart of the study.

**
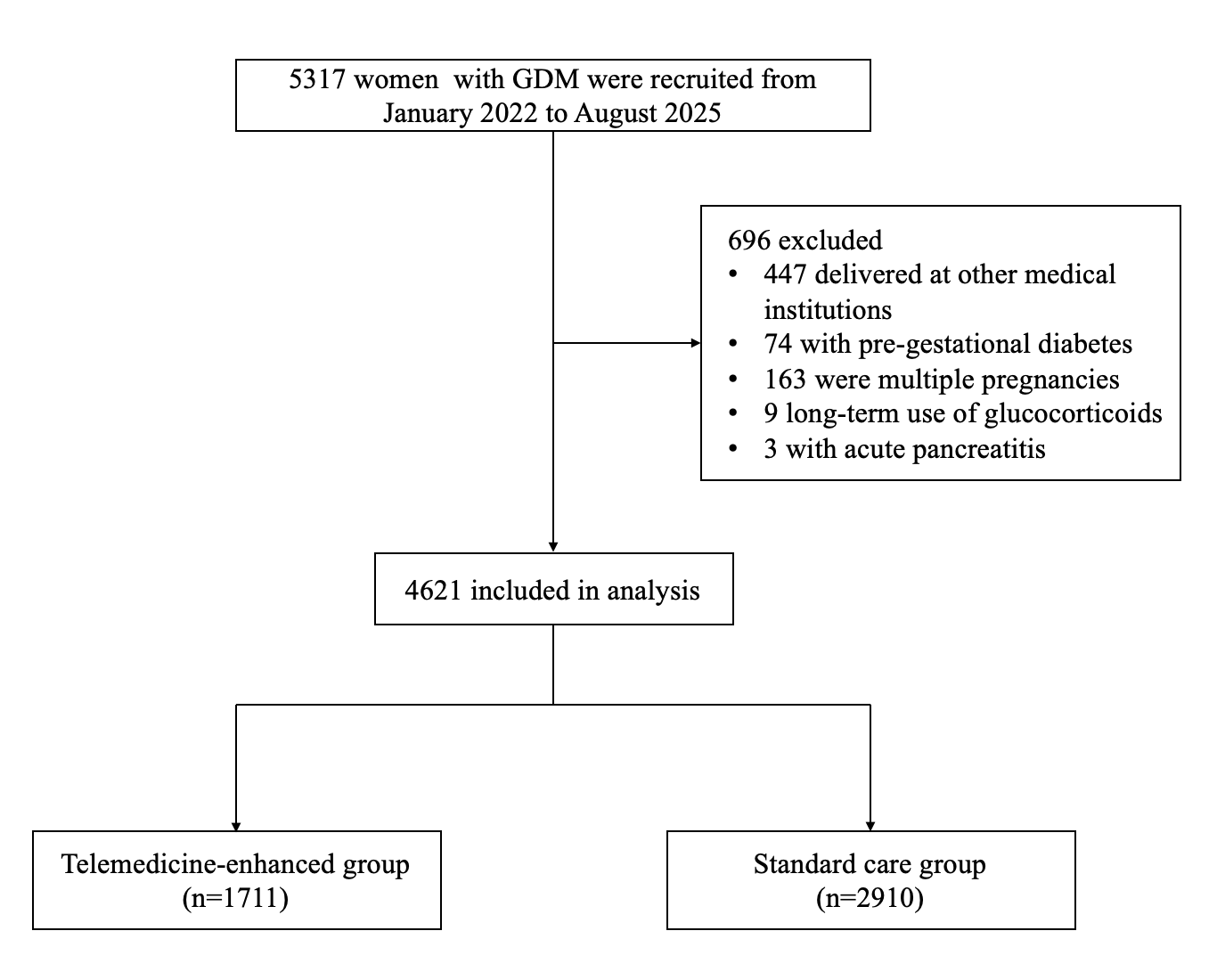
**

Abbreviations: GDM, gestational diabetes mellitus; EMR, electronic medical records.

**Figure S4.** Mediation analyses of (A) GWG and (B) mean FPG in the third trimester between telemedicine-enhanced care and various pregnancy outcomes.

**
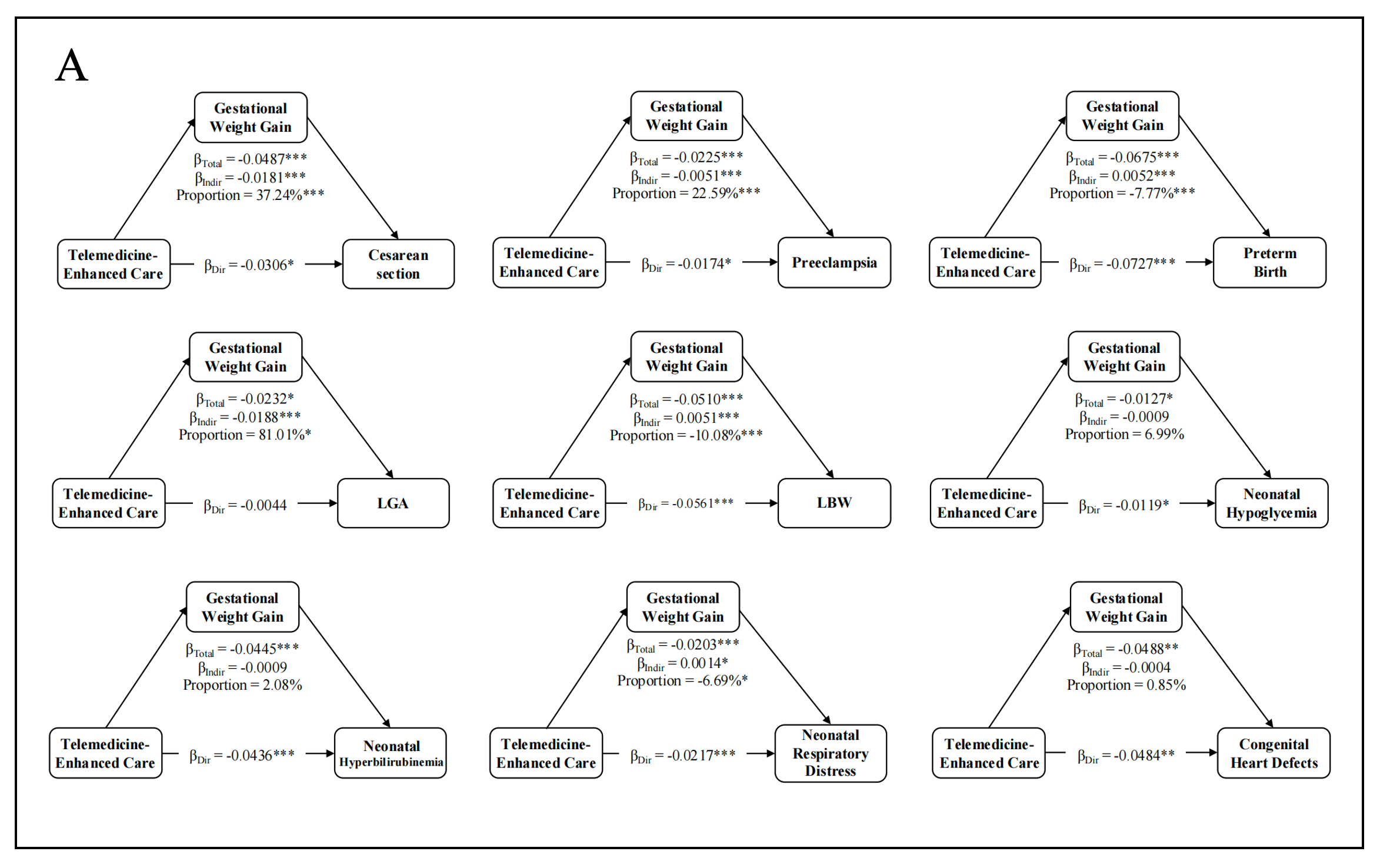
**

**
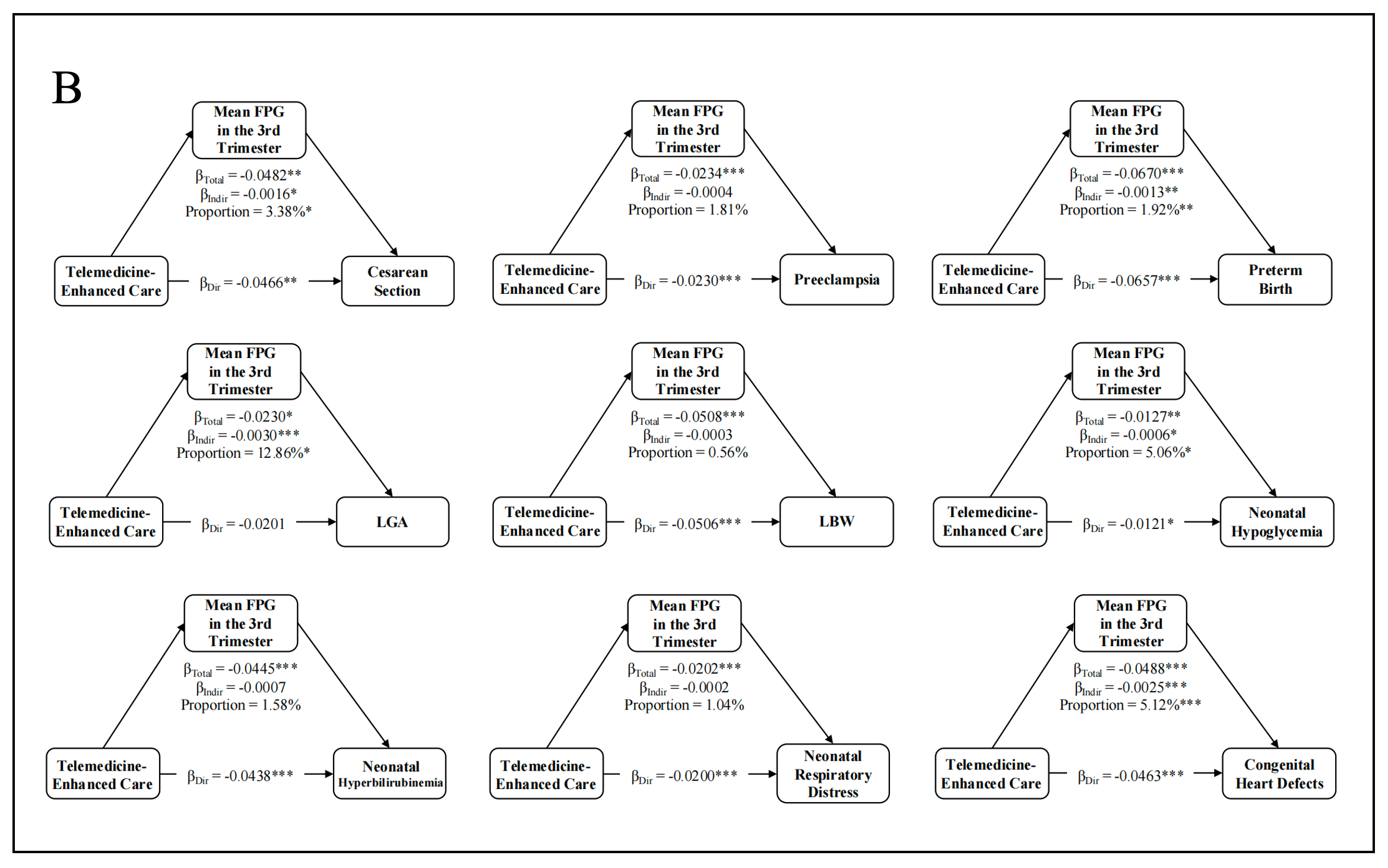
**

Abbreviations: GWG, gestational weight gain; FPG, fasting plasma glucose; LGA, large-for-gestational-age; SGA, small-for-gestational-age; LBW, low birth weight. ****P* value < 0.001, ***P* value < 0.01, **P* value < 0.05
